# Dual-Functional Triphenyltriindole “Knitting Polymers” for Efficient Iodine Capture and Zn–I_2_ Batteries

**DOI:** 10.1021/acsaenm.5c00709

**Published:** 2025-11-13

**Authors:** Nayara Méndez-Gil, Paula García-Balaguer, Lidia Martínez, Yves Huttel, Mohammad Afsar Uddin, María Luisa Ferrer, Berta Gómez-Lor

**Affiliations:** † Instituto de Ciencia de Materiales de Madrid (ICMM), CSIC, C/Sor Juana Inés de la Cruz 3, Madrid 28049, Spain; ‡ Escuela de Doctorado, Universidad Autónoma de Madrid, Madrid 28049, Spain

**Keywords:** truxene-based polymers, Friedel−Crafts reaction, adsorption, iodine, zinc−iodine batteries

## Abstract

This work introduces a class of nitrogen-rich porous polymers synthesized via “knitting polymerization” using a redox-active triphenyltriindole monomer. Two synthetic routes-thermal Friedel–Crafts reaction (**TRIPh-d**) and solvent-free mechanochemical activation (**TRIPh-m**) yield polymers with similar chemical structures but markedly different surface areas. Despite this, both materials exhibit exceptional iodine uptake from hexane solution (up to 1.87 g g^–1^), placing them among the highest-performing amorphous microporous organic polymers reported to date. The superior adsorption is attributed to the reversible oxidation of triindole units, forming radical cations that enhance iodine capture through electrostatic interactions. Comparative analysis with a truxene-based analog (**TX-m**) confirms the critical role of nitrogen-rich scaffolds over surface area alone. Beyond iodine sequestration, **TRIPh-d** also demonstrates outstanding performance as a cathode material in zinc–iodine batteries (ZIBs), delivering a specific capacity of 228 mA h g^–1^ at 1 A g^–1^, 99% Coulombic efficiency, and 72% capacity retention over 10,000 cycles. This dual functionality—combining environmental remediation with energy storage-along with the sustainability of the synthesis, positions these redox-active knitting polymers as promising candidates for future applications.

## Graphical abstract

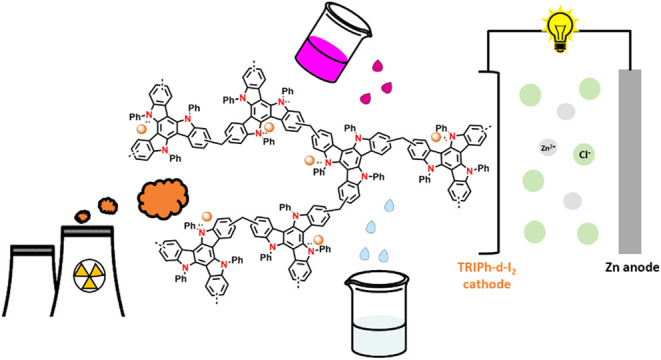

## 1 Introduction

Iodine adsorption is a critical process with wide-ranging applications across multiple sectors, including environmental protection, industrial processes, healthcare, and energy storage. In environmental contexts, effective adsorption techniques are essential for capturing and immobilizing radioactive iodine isotopes ^129^I and ^131^I, with half-lives of 8 days and 15.7 million years, respectively. These radionuclides, released primarily from the nuclear energy industry, pose significant environmental and health risks if they enter the food chain, potentially causing severe metabolic disruptions and cytotoxic effects such as thyroid cancer in humans.^1^ Beyond containment, iodine adsorbents are also being explored as platforms for the controlled release of iodine, capitalizing on its disinfectant properties or in the construction of advanced metal/I_2_ battery systems, such as zinc–iodine batteries (ZIBs), which takes advantage of the electroactive character of iodine confined within the adsorbent to store energy.^2,3^ All these diverse applications require different adsorption mechanisms to achieve optimal performance. For instance, long-lived radioactive isotopes such as ^131^I, require permanent immobilization within well-fitted porous spaces to prevent radiological release while an easy-release of the porous structure is required to take advantage of nonradioactive iodine antibacterial properties.^4,5^ In the case of ZIBs,^6^ an ideal adsorbent must be chemically stable and strike a balance between iodine confinement and ion accessibility. This is crucial because the inherently sluggish redox kinetics of the I^–^/I_2_ conversion and the shuttle effect of polyiodides (which can lead to anode corrosion), significantly limit battery lifespan and hinder practical application.^2,7^ In fact, for this application, the weak physical interactions between polyiodides and conventional carbon adsorbents offer only limited effectiveness in preventing the dissolution and diffusion of polyiodide species, despite their widespread use.^7^

Iodine adsorption is strongly influenced by the porous structure and the availability of active sites, which interact with iodine through mechanisms such as Lewis acid–base, coordinative or Coulomb interactions, hydrogen bonding···.^8^ In recent years, heteroatom doped carbons and high surface area porous organic polymers^9^ particularly those containing heteroatoms, such as O, N, S, etc.,^10–14^ which act as adsorption points, have emerged as promising alternatives for radionuclide sequestration and ZIBs. While the precise control of the pore size and heteroatom content in doped carbon remains challenging, the regular and accessible porous structures of crystalline covalent organic frameworks (COFs) offer significant advantages in terms of chemical species diffusion through their porous structures,^15^ making them particularly attractive for achieving remarkable iodine and radionuclide adsorption capacities^16^ as well as excellent performance in ZIBs.^17,18^ However, achieving crystallinity requires the reversible condensation of functional building blocks often via complex synthetic approaches.^19^ Furthermore, the reversible nature of the linking bonds, makes them much less stable than their amorphous counterparts (POPs). Unlike COFs, POPs are amorphous, highly cross-linked materials formed via the irreversible coupling of organic monomers through strong covalent bonds via multiple synthetic protocols ranging from metal-mediated couplings (Suzuki, Yamamoto, Sonogashira, Heck···) to acid or base-catalyzed polycondensation reactions or trimerizations, from conveniently functionalized monomers.^20,21^ This structural robustness imparts intrinsic porosity and exceptional thermal and chemical stability, making POPs highly suitable for environmental remediation,^22^ radionuclei sequestration^23^ and as cathode materials in ZIBs^16^

To advance toward real-life applications, easy and scalable synthetic protocols are essential. In this context,“*knitting polymerization*” ^24,25^ stands out as a particularly attractive strategy, as it involves the hyper-cross-linking of electron-rich monomers in the presence of halogenated solvents and Lewis acid catalysts.^26^ This method yields stable, high-surface-area porous polymers. A major advantage of this approach is that it does not require prior functionalization of the monomers or the use of expensive metal catalysts, which are commonly employed in most common cross-coupling polymerization methods.

In this synthetic approach, halogenated solvents not only serve as reaction medium but also act as linkers, “knitting” the electron-rich monomers together, via Friedel–Crafts reactions.^27^ To avoid the use of toxic solvents and the generation of large amounts of hazardous waste, alternative solvent-free mechanochemical synthesis that use only stoichiometric amounts of solvent have been developed.^28–31^ Continuous mixing of the reagents in a ball mill leads to the generation of highly reactive surfaces, significantly shortening the synthesis time and thus improving not only the environmental sustainability, but also the economic viability of the process.^32^

This manuscript introduces a “knitting polymer” based on a redox-active triphenyltriindole monomer,^33^ synthesized via thermal and mechanical Friedel–Crafts reactions (**TRIPh-d** and **TRIPh-m** respectively) and explores its capability in iodine adsorption for different applications. 10,15-Dihydro-5*H*-diindolo­[3,2-*a*:3′,2′-*c*]­carbazole (also known in the literature as triindole or triazatruxene) is a heptacyclic platform with remarkable p-type semiconducting properties, which has been extensively employed in the field of organic electronics.^34–36^ Owing to their favorable redox and electronic properties, triindole-based porous polymers have been also successfully used in a number of different applications, including photocatalysts,^37^ battery cells^38^ and high performance supercapacitors.^39^

The two different synthetic approaches (either under thermal or mechanical activations) resulted in polymers with a similar chemical structure but significant differences in surface area. Despite these variations, both polymers exhibit strong iodine adsorption from hexane solutions. This high adsorption capacity is attributed to the reversible oxidation of triphenyltriindole monomers, which form radical cations that enhance iodine adsorption through electrostatic interactions. The crucial role of redox-active nitrogenated positions in iodine removal has been confirmed by comparing the iodine uptake of these materials with that of a porous polymer, **TX-m**, made from the closely related monomer hexamethyltruxene. Despite its higher surface area (1025 m^2^·g^–1^), **TX-m** demonstrated lower adsorption capability. Truxene, which has the same heptacyclic geometry as triindole but is composed solely of carbon and hydrogen, gives rise to polymers with significantly lower HOMO values, making them more difficult to oxidize.^40^ These differences in adsorption performance were more pronounced in solution than in vapor, suggesting that the oxidation-driven adsorption process is favored in solution.

Moreover, the ability of **TRIPh** to effectively incorporate and retain iodine within its porous structure has proven advantageous in balancing iodine diffusion and retention in ZIBs. Interestingly, **TRIPh-d** has demonstrated high performance as a cathode material, exhibiting high specific capacity, stable Coulombic efficiency, and strong capacity retention. These results highlight dual functionality of this materials for both environmental remediation and sustainable energy storage applications.

In summary, this work presents a significant advancement in the development of multifunctional porous polymers by integrating redox-active nitrogen-rich scaffolds into knitting polymer architectures. The dual functionality of this material, along with the scalability and sustainability of the synthesis, positions these knitting polymers as promising candidates for future applications in clean energy and environmental technologies.

## 2 Experimental Section

All the details regarding the chemicals, characterization techniques, photocatalytic experimental setup and electrochemical measurements are given as Supporting Information.

### 2.1 Synthesis of **TRIPh-d**

A mixture of *N*-triphenyltriindole (200 mg, 0.35 mmol), AlCl_3_ (600 mg, 4.5 mmol) in 14 mL of CH_2_Cl_2_ was stirred at 40 °C for 16 h. The mixture was poured on water and stirred for 30 min then the solid formed was filtered and washed with acetone, water and MeOH. The solid was suspended in a HCl (1N): MeOH 1:1 solution for 30 min, filtered and subsequently suspended in a solution NH_3_:MeOH 1:1 solution overnight and dried under vacuum at 200 °C for 16 h to render **TRIPh-d** as a brown powder (116 mg, 56% yield). Elemental Analysis Calcd for C_43.5_H_27_N_3_: C 87.94, H 5.14, N 6.91. Found: C 79.05, H 4.68, N 5.68.

### 2.2 Synthesis of **TRIPh-m**

A mixture of *N*-triphenyltriindole (200 mg, 0.35 mmol), AlCl_3_ (600 mg, 4.5 mmol) and 0.3 mL of CH_2_Cl_2_ was introduced into a ball-mill, and grinded for 3 h at 500 rpm. After this time, the mixture was suspended in water and stirred for 30 min and subsequently filtered and washed with acetone, water and MeOH. The solid was suspended in a HCl (1N): MeOH 1:1 solution for 30 min, filtered and subsequently suspended in a solution NH_3_:MeOH 1:1 solution overnight and dried under vacuum at 200 °C for 16 h to render **TRIPh-m** as a brown powder (128 mg, 62%). Elemental Analysis Calcd for C_43.5_H_27_N_3_: C 87.94, H 5.14, N 6.91. Found: C 79.32, H 4.21, N 5.91.

### 2.3 Synthesis of **TX-m**

A mixture of hexamethyltruxene (200 mg, 0.47 mmol), AlCl_3_ (690 mg, 5.2 mmol) and 0.3 mL of CH_2_Cl_2_ was introduced into a ball-mill, and grinded for 3 h at 500 rpm. After this time, the mixture was suspended in water and stirred for 30 min and subsequently filtered and washed with acetone, water and MeOH. The solid was suspended in a HCl (1N): MeOH 1:1 solution for 30 min, filtered and subsequently suspended in a solution NH_3_:MeOH 1:1 solution overnight and dried under vacuum at 200 °C for 16 h to render **TX-m** as a brown powder (191 mg, 93%). Elemental Analysis Calcd for C_34.5_H_30_: C 93.2, H 6.80. Found: C 84.01, H 5.84.

## 3 Results and Discussion

### 3.1 Materials Preparation and Characterization

The synthesis of the polymers is depicted in Scheme 1. All three polymers were obtained by the Friedel–Crafts reaction of 200 mg of the corresponding truxene or triindole monomer, in the presence of an excess of AlCl_3_. **TRIPh-d** was obtained by refluxing this mixture in CH_2_Cl_2_ (14 mL) at 40 °C for 16 h (Method A). **TRIPh-m** and **TX-m** were obtained by mechanically grinding this mixture for only 3 h, in the presence of traces of CH_2_Cl_2_ (Method B), making this synthetic approach more environmentally friendly. After filtration and thorough washing of the polymer with water, ammonium, and organic solvents, the three polymers were obtained as brown powders in 56, 62 and 92% yield, respectively. Note that the synthesis of **TX-m** have been previously reported via thermal activation, rendering a high surface area polymer with interesting photocatalytic activity.^41,42^ Under these conditions, the solvent molecules act not only as the reaction medium but also as linkers for the electron-rich triindole and truxene platforms. It is important to emphasize that although the nitrogen-attached phenyl groups in **TRIPh-m** could theoretically participate in the reaction, this possibility was ruled out through control experiments. Specifically, we studied the reaction using nonanoyl chloride as the electrophile to generate a soluble model compound, which confirmed that the phenyl groups remain unreactive under the given conditions (Figure S1).

**Scheme 1:**
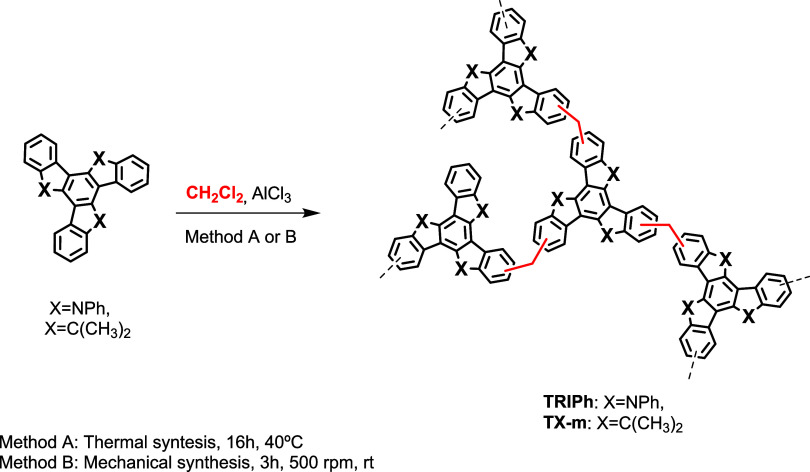
Synthesis of Porous Polymers **TRIPh-d, TRIPh-m** and **TX-m**

The chemical structures of **TRIPh-d, TRIPh-m** and **TX-m**, were investigated by means of FT-IR, ^13^C NMR and elemental analysis. The FT-IR spectra of **TRIPh-d** and **TRIPh-m** (Figure 1a) exhibit a series of characteristic peaks between 1595 and 1300 cm^–1^ related to C=C stretches of the aromatic rings of the triphenyltriindole units and a peak at 1220 cm^–1^ attributed to C–N absorption. On the other hand, **TX-m**, presents and a number of peaks between 1610 and 1350 cm^–1^ typical of the truxene skeleton vibrations and characteristic peaks at ∼3020–2920 cm^–1^ attributed to ν­(C–H) vibrations of the methyl and methylene groups.

**Figure 1:**
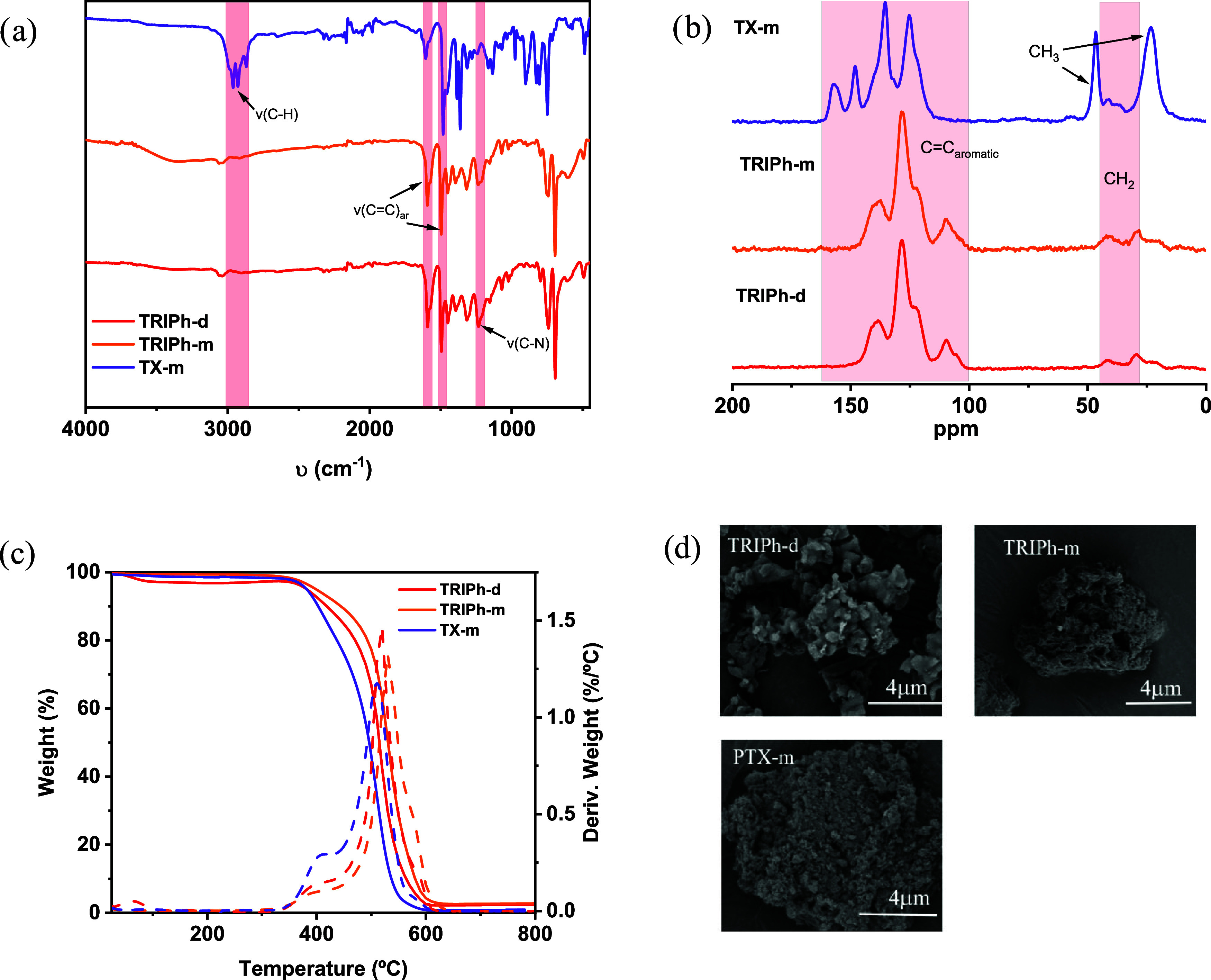
(a) FT-IR spectra, (b) ^13^C NMR spectra, (c) TGA and (d) SEM images of **TRIPh-d**, **TRIPh-m** and **TX-m**.

The solid ^13^C CP-MAS NMR spectrum of **TX-m** (Figure 1b) exhibits two peaks at 20–60 ppm attributed to methylene carbons (i.e., from truxene and linker). In the aromatic area, 4 peaks can be observed between 115 and 160 ppm corresponding to the aromatic carbon of truxene. Likewise, the solid ^13^C CP-MAS NMR spectrum of **TRIPh-d** and **TRIPh-m** presents two peaks between 23 and 47 ppm corresponding to the linker and 3 peaks between 100 and 150 ppm which correspond to the aromatic carbon of triindole and phenyl moieties.

The thermal stabilities of **TRIPh-d**, **TRIPh-m**, and **TX-m** porous polymers were studied using Thermogravimetric Analysis (TGA), as illustrated in Figure 1c. The three polymers demonstrate thermal stability up to 380 °C.

Elemental analyses of the polymers reveal slightly lower percentage values for C, H, and N as compared to the calculated values probably due to trapped solvent (Table S1) as commonly observed in porous organic polymers.^43^ All polymers exhibit broad diffraction peaks in their PXRD (Figure S3), consistent with the expected disordered amorphous structures characteristic of kinetically controlled processes. Field-emission scanning electron microscopy (SEM) images of the polymers are characterized by aggregates of spherical structures (Figure 1d) showing the typical morphology of porous polymers.

The surface area and pore size distribution for these porous polymers were determined by measuring the nitrogen adsorption–desorption isotherms at 77 K. The accessible surface areas, determined using Brunauer–Emmett–Teller (BET) theory, are 520 m^2^ g^–1^ for **TRIPh-d**, 54 m^2^ g^–1^ for **TRIPh-m**, and 1025 m^2^ g^–1^ for **TX-m** (Figure 2a). Note that while the Elemental Analysis, FTIR, and ^13^C NMR spectra of **TRIPh-d** and **TRIPh-m** are nearly identical indicating that they have the same chemical structure, significant differences are observed in terms of surface areas. A comparable phenomenon can be observed in the case of **TX-m**, which has the same chemical structure as that previously synthesized via thermal activation but exhibits a slightly reduced surface area.^42^ The total pore volume at *p*/*p*_0_ = 0.99 was found to be 0.32, 0.05 and 0.85 cm^3^ g^–1^ for **TRIPh-d, TRIPh-m** and **TX-m** respectively. The pore size distribution of the three polymers, calculated using nonlocal density functional theory (NLDFT), confirms the presence of both micropores and mesopores. The mesopores are mainly concentrated at 2.2 and 2.6 nm for **TRIPh-m** and **TRIPh-d**, respectively, and at 2.8 nm for **TX-m** (Figure 2b). These pore sizes are large enough to allow iodine molecules to enter easily, but small enough to maintain strong adsorption interactions.

**Figure 2:**
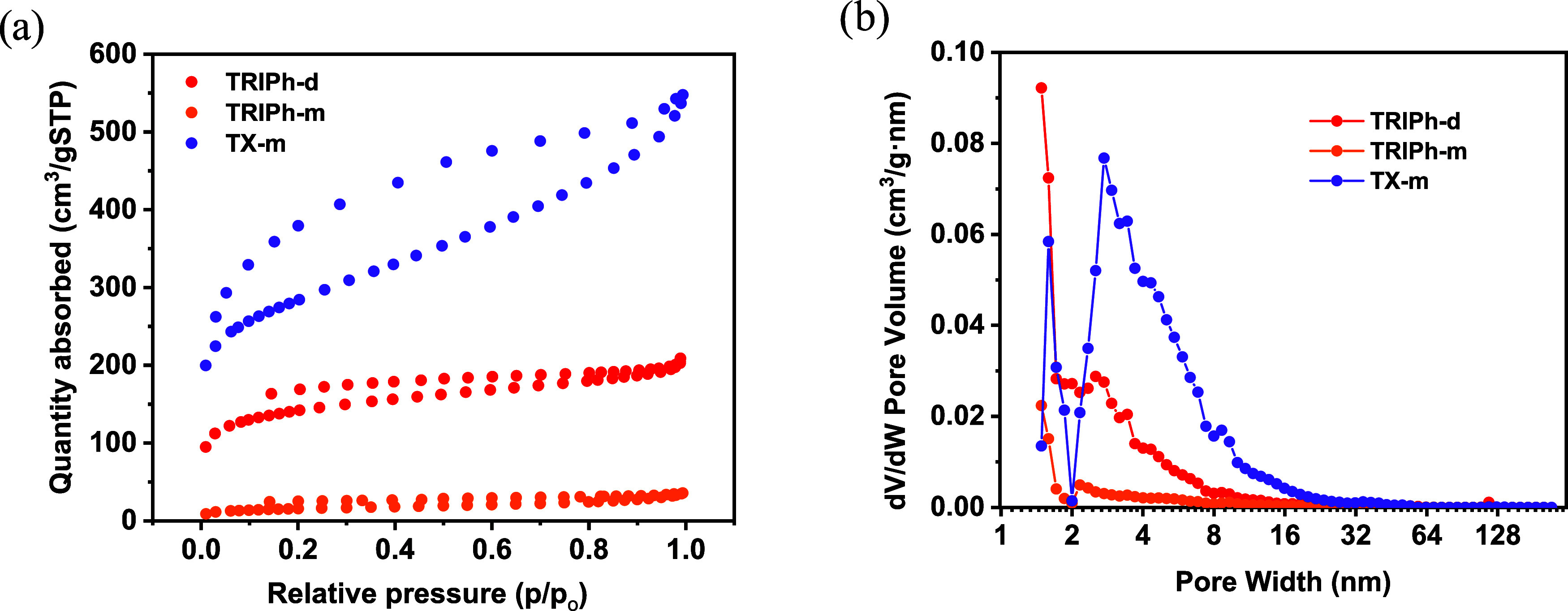
(a) Adsorption/desorption isotherms and (b) pore size distribution of **TRIPh-d**, **TRIPh-m** and **TX-m**.

### 3.2 Iodine Capture in **TRIPh-d, TRIPh-m** and **TX-m** POPs

The iodine uptake capacity of these polymers was evaluated by gravimetric measurements. Twenty-five mg of each polymer were preweighed and introduced in a closed vessel containing a vial with 1 g of iodine at 75 °C and atmospheric pressure. Iodine uptake was determined by measuring the weight increase of the different solids at various time intervals (Figure 3a). The samples showed a significant increase in iodine uptake within the first 24 h. After 24 h, the weight variations were negligible indicating that the adsorption equilibrium is reached.

**Figure 3:**
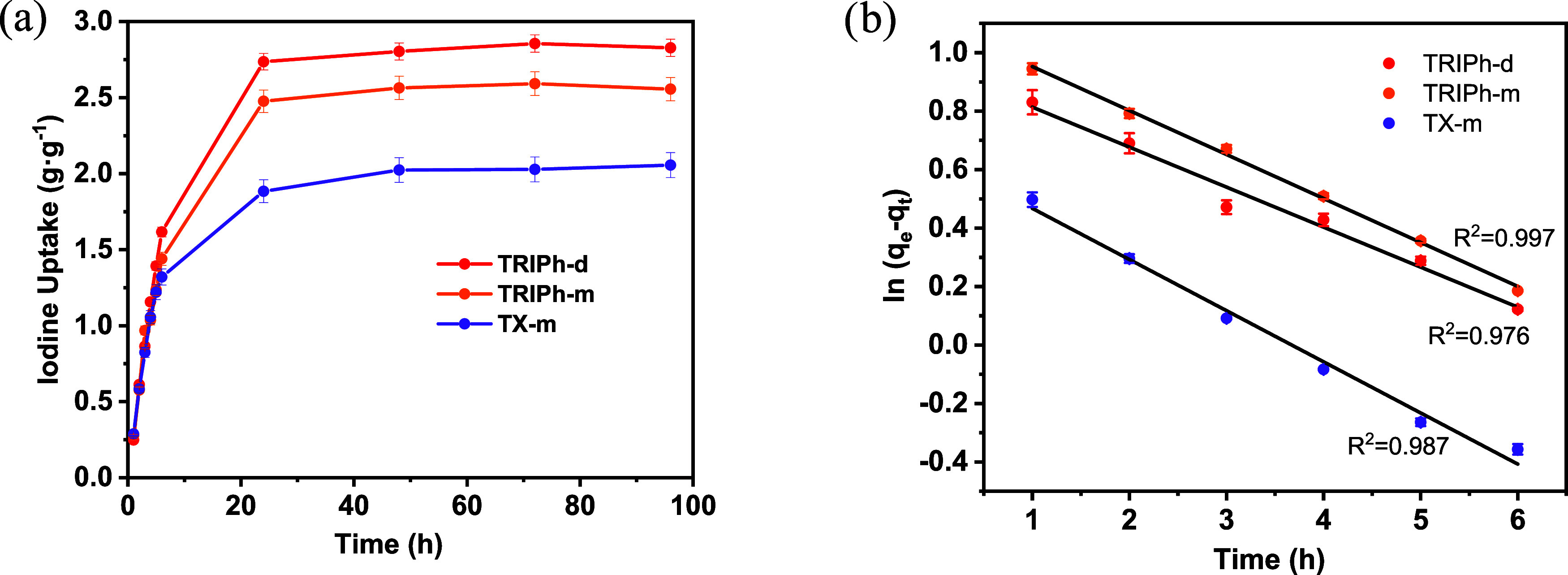
(a) Vapor iodine uptake and (b) adsorption kinetics of polymers **TRIPh-d** (red), **TRIPh-m** (orange) and **TX-m** (purple) estimated error <5%.

All three polymers demonstrate substantial iodine uptake capacities, with adsorption performance trends confirming that both accessible surface area and heteroatom content critically impact vapor iodine adsorption. The nitrogen-rich **TRIPh-d** exhibited the highest iodine uptake (282 wt %), followed by **TRIPh-m** (257 wt %), while **TX-m** showed the lowest adsorption capacity (202 wt %), despite having the largest surface area, highlighting the key role of nitrogen atoms as active adsorption sites. The superior performance of **TRIPh-d** over **TRIPh-m** can be attributed to its larger surface area and larger mesopores, which will facilitate iodine vapor diffusion and improve access to nitrogen functionalities. Interestingly, iodine-release monitoring in ethanol solution revealed that after 1 h, 99% of the iodine adsorbed in **TX-m** was released, while **TRIPh-d** and **TRIPh-m** exhibited significantly lower release rates of 16 and 37%, respectively (Figure S7), further confirming the strong binding of iodine in the nitrogen-rich framework.

The adsorption kinetics were analyzed using pseudo-first and pseudo-second order models. In all three cases, the adsorption kinetics are better described by the pseudo-first order model, suggesting that also the diffusion across the adsorbent surface is a key determinant of adsorption rate (Figure 3b and Table S3). While **TX-m** has a lower overall adsorption capacity, the pseudo-first-order model fitting indicates that it reaches equilibrium more rapidly, resulting in a higher calculated rate constant. This can be rationalized by considering its significantly larger surface area and the presence of more accessible mesopores, which likely facilitate faster diffusion of iodine vapor throughout the polymer matrix. These structural features contribute to a quicker adsorption rate, even though the total iodine uptake is lower compared to **TRIPh-d** and **TRIPh-m**.

The polymers were fully regenerated using a 48-h methanol wash in a Soxhlet extractor and subsequently tested over multiple adsorption–desorption cycles. Notably, **TX-m** retained 98% of its adsorption efficiency after five cycles (Figure S7), while **TRIPh-d** and **TRIPh-m** retained 84 and 96%, respectively. The slight decreases in performance for **TRIPh-d** and **TRIPh**-m are likely due to the presence of strongly bound iodine species at nitrogen sites, which limit complete regeneration of the polymer network during washing.

We have also investigated the ability of these polymers to adsorb iodine from an *n*-hexane solution. Interestingly, in this case, much more notable differences were observed when comparing the adsorption behavior of the nitrogen-containing polymers, **TRIPh-d** and **TRIPh-m**, to that of **TX-m**, which contains only carbon and hydrogen. In these experiments, 5 mg of each porous polymer was added to 5 mL iodine solutions in hexane at varying concentrations and placed in the dark without stirring for 24 h. The concentration of remaining iodine was determined by UV–vis absorption.

The adsorption capacity of the iodine at equilibrium (*Q*_e_) was investigated as a function of equilibrium concentrations (*C*_e_) following the eq 1, as illustrated in Figure 4a.

1 $$Q_{e}=\frac{C_{i}-C_{e}}{M}\cdot V$$

where *Q*_e_ is the amount of the iodine adsorbed by the porous polymer (mg g^–1^), *C_i_* and *C*_e_ the initial and the equilibrium concentrations of the iodine solution, *M* the weight of adsorbent (g) and *V* the working volume on liter (L).

**Figure 4:**
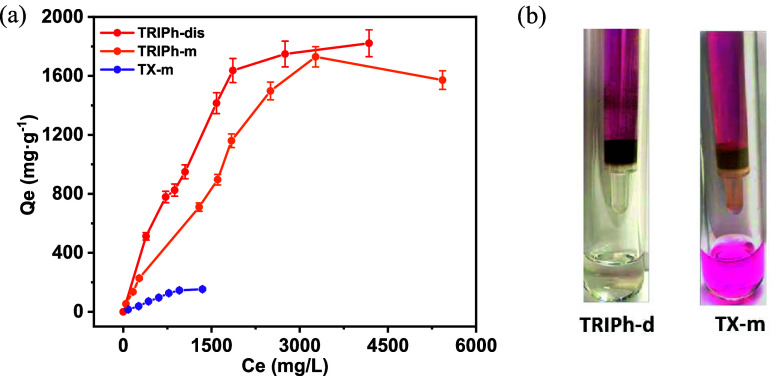
(a) Isotherm study of iodine adsorption in solution by **TRIPh-d**, **TRIPh-m**, and **TX-m.** Estimated error <5%. (b) Photographs of **TRIPh-d** (left) and **TX-m** (right) during filtration of a 300 mg L^–1^ iodine solution in hexane.

The maximum iodine uptake capacity estimated for **TRIPh-d**, is 1817 mg g^–1^ which to our knowledge represents the highest uptake capacity reported to date for an organic porous polymer (Table S4). In spite of its low surface area a very high value was also found for **TRIPh-m** (1530 mg g^–1^), while the adsorption capacity of **TX-m** was only of 53 mg g^–1^ (Figure 4a).

To gain a deeper insight into the adsorption process, the equilibrium data of iodine adsorption were analyzed using Langmuir and Freundlich isotherm models^44^ (Figures S9, S10 and Table S5). The correlation coefficients obtained of the Freundlich isotherm model (RF: 0.97, 0.99, and 0.99), are higher than those of the Langmuir isotherm model (RL: 0.87, 0.84 and 0.54) suggesting that the adsorption in solution of iodine is multilayered and heterogeneously adsorbed on the surface of the polymer.^45^

In order to progress toward practical applications and considering the high adsorption capacity exhibited by **TRIPh-d** we have evaluated the iodine removal efficiency by passing the iodine solution through a column of the polymer. To that goal 40 mg of **TRIPh-d** was loaded into an empty cartridge and compacted mechanically. Subsequently, 30 mL of a 300 mg/L iodine solution in hexane was passed through the column by gravity (Figure 4b left) and the amount of iodine remaining in the filtrate was determined by UV absorbance. A removal efficiency of 89% within just 120 min, could be achieved. The column was washed by passing methanol with a little amount of ammonium hydroxide and the experiment was repeated. After 3 adsorption/washing cycles, the polymer **TRIPh-d** maintained a removal efficiency of 87%. This experiment holds a great promise toward its practical applications.

Interestingly performing this experiment using the polymer **TX-m** resulted in only a removal of an 8% of a 30 mL solution with the same concentration (Figure 4b right).

The adsorption kinetics of iodine on **TRIPh-d** was analyzed using both pseudo-first-order and pseudo-second-order models. Unlike iodine adsorption in vapor, the pseudo-second-order model provided a better fit (Figure S12 and Table S6), suggesting that chemisorption is the predominant interaction between the adsorbent and the adsorbate. This chemisorption process likely involves a redox reaction in which iodine interacts with easily oxidizable nitrogen sites on the polymer. The redox-active nature of the triphenyltriindole monomers likely enhances this interaction, helping to explain the substantial differences in adsorption behavior compared to **TX-m**.

Additional adsorption studies were conducted in aqueous iodine solutions to evaluate the robustness and selectivity of **TRIPh-d** under conditions relevant to nuclear wastewater (see section 6 in the Supporting Information). The maximum adsorption capacity of **TRIPh-d** in aqueous solution remained remarkable (819 wt %), although lower than in hexane, as expected due to the reduced proportion of oxidizing molecular iodine in water. Nevertheless, **TRIPh-d** exhibited consistent adsorption behavior across a range of pH values (Figure S15) and in the presence of interfering ions (Cl^–^, NO_3_^–^, and CO_3_^2–^). These results, together with aqueous-phase isotherm data and selectivity tests, support the material’s potential for iodine removal in complex aqueous environments. A full evaluation under realistic nuclear wastewater conditions will be addressed in future work.

We have analyzed the iodine-loaded **TRIPh-d** polymer by PXRD, IR and UV–vis spectroscopy (Figures S16–S18). No characteristic peaks of elemental iodine were observed in the PXRD spectra (Figure S16), suggesting that iodine did not form localized crystalline aggregates, resulting instead in a uniform distribution throughout the polymer.^46^ The FTIR spectra (Figure S17) showed significant broadening on the aromatic stretching bands associated with the triindole skeleton as well as on the peak at 1220 cm^–1^ attributed to C–N stretching. This broadening indicates a strong interaction between iodine and the monomers. Such pronounced changes support the formation of a cation radical, which, as previously calculated, is delocalized across the aromatic platform.^47^ The UV–vis spectrum of the iodine-loaded polymer show a significant red shift compared to that of the bare polymer (Figure S18), suggesting charge transfer interactions. Peaks at 298 and 368 nm (attributed to iodine I_3_^–^) and at 486 nm (attributed to I_2_) are also detectable.

To shed light into the mechanism of the iodine capture process and to understand the differences observed in adsorption performance of various polymers in both vapor and solution phases, the nature of the adsorbed iodine species was investigated using XPS and Raman spectroscopy.

XPS of the three polymers saturated with iodine vapors confirmed the presence of iodine, evidenced by the presence of 3d_5/2_ and 3d_3/2_ peaks. Figure 5a displays the analysis of the I 3d core level spectra of the two peaks together with the resulting deconvolution of the peaks. The fitting has been applied to both 3d_5/2_ and 3d_3/2_ core levels while keeping constant the spin–orbit coupling energy shift at 11.5 eV^48^ and the intensity ratio 2/3.^49^ Note that although the discussion of the results will be performed on the I 3d_5/2_, it holds also for the I 3d_3/2_ components. The asymmetry of the peaks clearly indicated the presence of at least three components: one at 618.9 ± 0.1 eV, a second at 620.8 ± 0.1 eV and a smaller component at 624 ± 0.2 eV, which suggests the presence of iodine in form of I_3_^–^ (618.9 eV) and I_2_ (620.8 eV). The third smaller component at higher BE is a shakeup satellite of [I_3_]^−^ compound. Considering the intensity of the different peaks, we can estimate the I_2_/I_3_^–^ ratio as 1.1 for **TRIPh-m**, 1.4 for **TRIPh-d**, and 2.2 for **TX-m** (Figure 5a). Accordingly, the largest proportion of adsorbed molecular iodine is found in **TX-m**, followed by **TRIPh-d** and **TRIPh-m**, which correlates with their respective surface area trends. This suggests that higher surface areas favor the diffusion of I_2_ vapors through the porous networks. For the nitrogen-containing polymers, we also investigated the N 1s core-level peak (Figure 5b). Interestingly, the bare **TRIPh** sample exhibited a major component at 400.5 ± 0.1 eV, consistent with previous reports on triindole-containing polymers.^50^ A secondary component was observed at 399.1 ± 0.1 eV, typically attributed to C=N bonds.^51^ We tentatively assign this second component to the presence of highly delocalized, oxidized triindole units, which may lead to C–N bond shortening within the heptacyclic framework, as supported by previous calculations.^47,52^ Upon iodine loading, the intensity of this second component increases approximately 3-fold, suggesting that diffused iodine can readily oxidize the triaryl-substituted nitrogen atoms, forming the corresponding cation radicals. This observation contrasts with the behavior typically seen in nitrogen-rich porous polymers, where nitrogen participates in charge transfer from the lone pair to iodine, usually resulting in a higher binding energy due to increased electron density around the nitrogen atoms.^51,53^ The positive charge of these radicals likely enhances electrostatic interactions with I_3_^–^ species generated during the sorption process, resulting in a higher proportion of I_3_^–^ in **TRIPh-d** and **TRIPh-m**.

**Figure 5:**
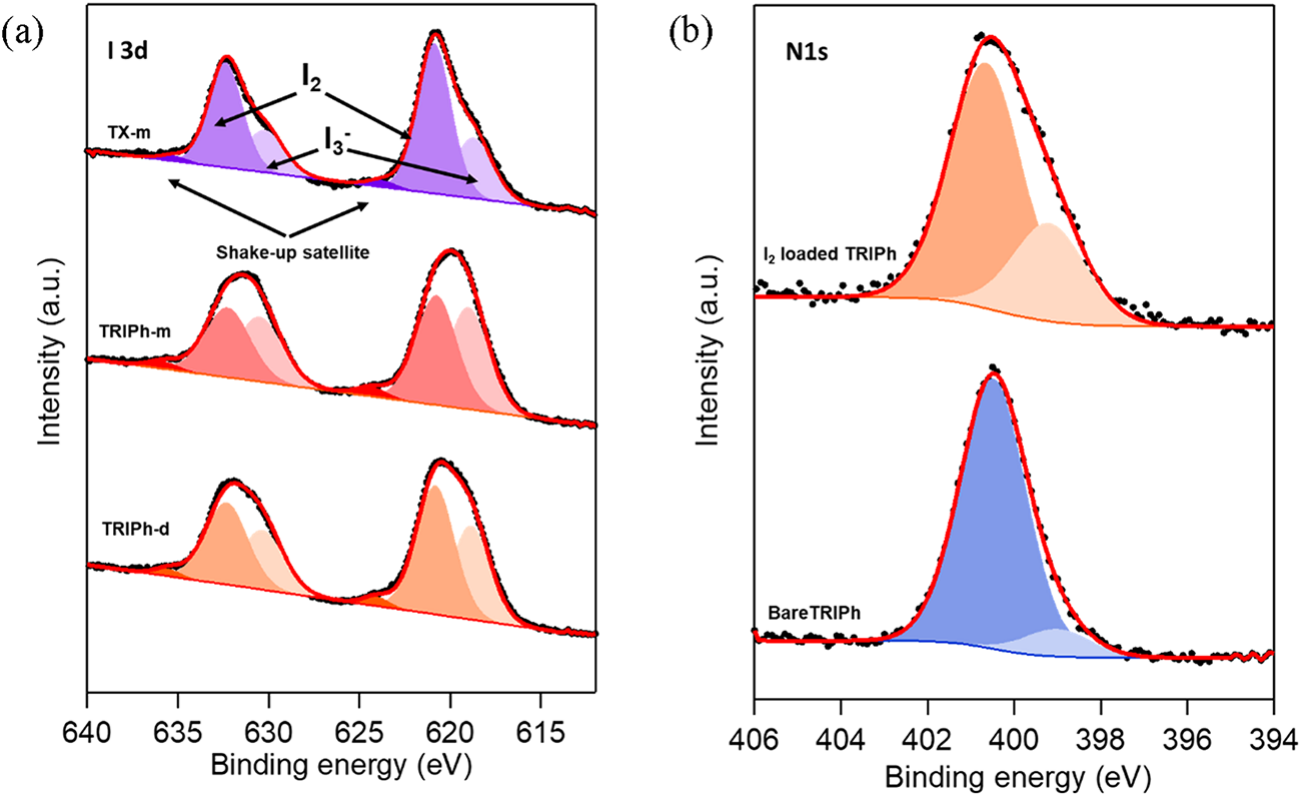
(a) I 3d XPS core level spectra of iodine loaded **TRIPh-m** (bottom), **TRIPh-d (**middle**)** and **TX-m** (top) polymers. (b) N 1s XPS core level spectra of bare (bottom) and iodine loaded **TRIPh**(top) polymer.

This strong interaction explains the difficulty in desorbing iodine once adsorbed. Interestingly, it may also provide an optimal balance between ion diffusion and retention, facilitating the reversible I_2_/I_3_^–^ conversion while effectively retaining I_3_^–^ within the polymer matrix, as required for ZIBs.

Figure 6 shows a comparison of the Raman spectra of the three different polymers after adsorption of iodine from solution or vapor. As can be observed, while the adsorbed iodine species in vapor show stretching vibrations associated with I_2_ (180 cm^–1^) and I_3_^–^ (111 cm^–1^),^54,55^ the iodine species adsorbed in solution consist mainly in polyiodides I_3_^–^ (111 cm^–1^) and I_5_^–^ (168 cm^–1^).^56–58^ Note that the to the low iodine uptake of this polymer from hexane solutions and the strong fluorescence of **TX-m** under Raman conditions, masks the characteristic signals of iodine species and prevents the detection of iodine from hexane solution **TX-m** (Figure 6c).

**Figure 6:**
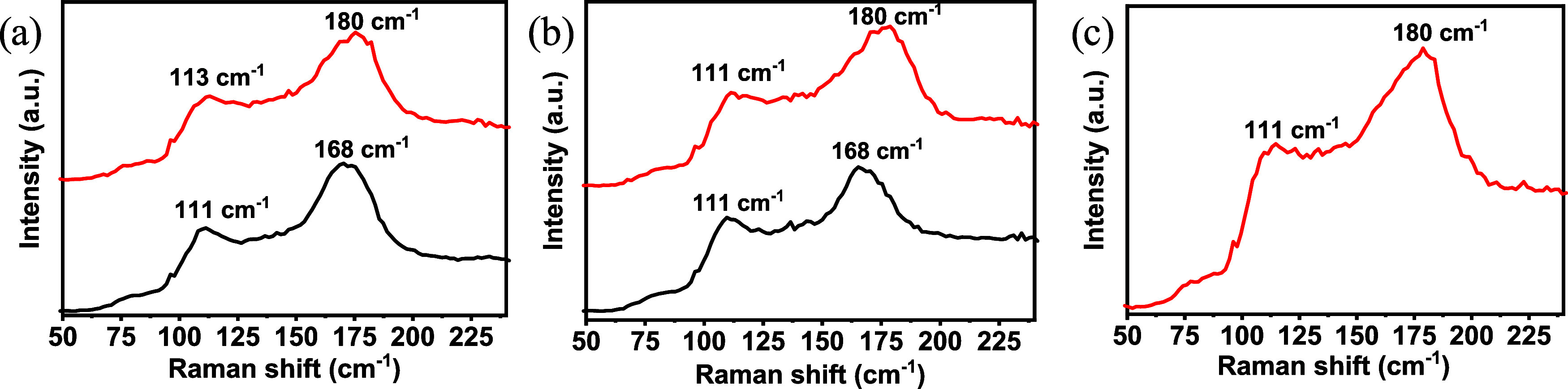
Raman spectra of (a) **TRIPh-d** (b) **TRIPh-m** and (c) **TX-m** after iodine adsorption in solution (black) and vapor (red).

The different nature of the adsorbed species explains the remarkable differences observed in the adsorption trends when comparing iodine adsorption in vapor to solution for the different polymers. In vapor, the high surface area of **TX-m** will facilitate the diffusion of iodine into the pore and its adsorption by the electron-rich surface thus explaining the slight differences in iodine adsorption observed in comparison to **TRIPh-d** and **TRIPh-m** containing nitrogen in the active site. However, in solution, the remarkable adsorption of iodine found for **TRIPh-d** and **TRIPh-m** is probably produced by redox reaction of iodine with the readily oxidable nitrogen sites, thus explaining the predominance of polyiodide species in the iodine-loaded species and the drastic differences in the maximum adsorption values when compared to **TX-m**.

### 3.3 I_2_ Loaded TRIPh POPs as Cathodes in Zn/I_2_ Batteries

Porous organic polymers (POPs) capable of retaining iodine species within the cathode have attracted significant interest for improving Zn/I_2_ battery performance. In these batteries, polyiodide intermediates (I_3_^–^, I_5_^–^) are formed during the reversible redox conversion between I^–^ and I_2_. However, the high solubility of these species can lead to an uncontrolled migration between electrodes, resulting in capacity loss, self-discharge, and reduced cycle life. **TRIPh** networks demonstrate superior iodine retention by stabilizing polyiodide species, offering a better balance between iodine diffusion and retention compared to **TX-m** POP. This makes them highly suitable for Zn/I_2_ battery cathodes. Iodine loading onto **TRIPh-d** and **TRIPh-m** was performed by immersion of the polymers in a *n*-hexane iodine solution (900 mg L^–1^) for 48 h, after which the resulting black solids were collected. The iodine contents of the two networks were determined by TGA, yielding loadings of 16.8 and 11.8% for **TRIPh-d** and **TRIPh-m**, respectively (Figure S19). For charge storage analysis, Swagelok cells were assembled using zinc foil as the anode, 30 m ZnCl_2_ as the electrolyte, a PVDF filter-paper as separator, and a free-standing composite composed of iodine-loaded polymer, carbon black, and PTFE binder as cathode (see details in Section S8.2 of Supporting Information).

Figure 7a show the cyclic voltammogram (CV) of iodine-loaded **TRIPh-d** and **TRIPh-m** in the potential range of 0.6–1.6 V vs Zn^2+^/Zn. A highly reversible redox peak was observed at 1.22 vs Zn^2+^/Zn corresponding to I^–^/I_2_ redox reaction within the polymer network. **TRIPh-d** shows a higher peak intensity than **TRIPh-m**, which translates in higher specific capacity. This is consistent with its higher iodine adsorption and its higher surface area.

**Figure 7:**
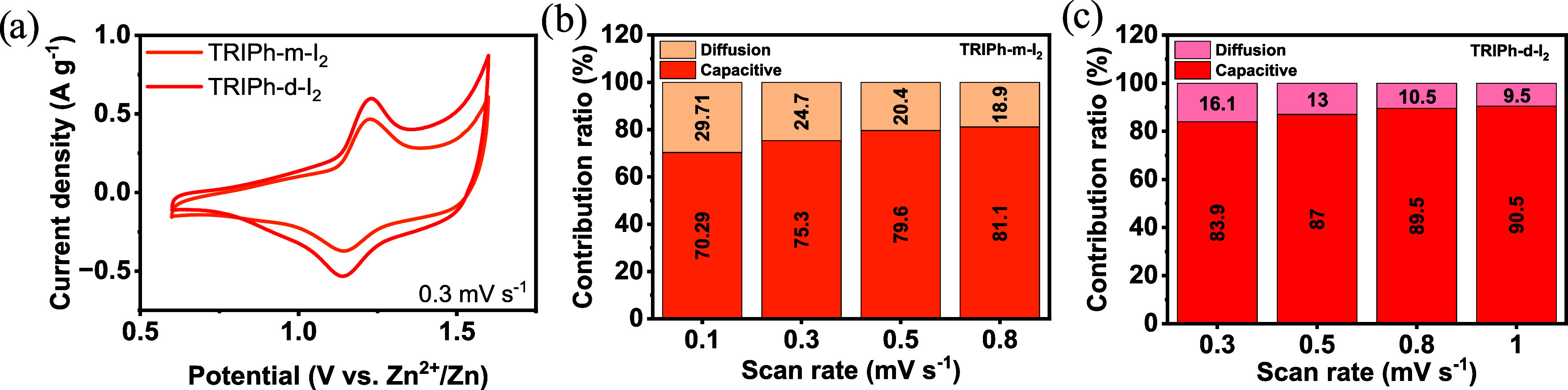
(a) CV curve at 0.3 mV s-1 in the potential range of 0.6–1.6 V of **TRIPh-m** and **TRIPhd** (b) Contribution ratios (%) of diffusion and capacitance of **TRIPh-m** (c) and **TRIPh-d**.

The CVs at various scan rates were further analyzed to evaluate the capacitive and diffusion-controlled contributions (Figure S20). The equation *i* = *av* ^*b*^ describes the relation between the peak current (*i*) and the scan rate (*v*); when the parameter b is close to 1, the process is capacitive-limited involving charge storage primarily on the electrode surface, and when it approaches 0.5, the process is diffusion-controlled. Analysis of the log­(*i*)–log­(*v*) plots (Figure S21) yielded *b*-values of 0.97 (oxidation) and 0.94 (reduction) for **TRIPh-d**, while the **TRIPh-m** network exhibited slightly lower values of 0.87 and 0.89, respectively. These results indicate that both polymer networks primarily operate via a capacitive charge-storage mechanism (electric double layer capacitance, EDLC, and pseudocapacitive adsorption), with **TRIPh-d** exhibiting the strongest capacitive contribution (Figure 7b,7c). This behavior is compatible with the adsorption of generated polyiodide species on the positive partially oxidized **TRIPh** networks, contributing to reversible charge storage. Moreover, increasing the scan rate further elevated the capacitive contribution in both networks, reflecting a higher proportion of surface-controlled processes.

Figure 8a,8b show the galvanostatic charge and discharge (GCD) curves of **TRIPh-d** and **TRIPh-m** showing plateaus corresponding to the oxidation and reduction process of I_2_. The measured capacity of **TRIPh-m** (157 mA h g^–1^ at 1 A g^–1^) corresponds to a 74% utilization of the iodine two-electron transfer. Not achieving the theoretical capacity of iodine for a two-electron transfer (211 mA h g^–1^) in this polymer is likely due to incomplete utilization of the iodine redox reaction, which can be limited by the low surface area of **TRIPh-m** and by restricted access to iodine sites. For **TRIPh-d**, the obtained capacity of 228 mA h g^–1^ at 1 A g^–1^, slightly exceeds the iodine theoretical capacity. As mentioned above, a large portion of the charge storage is surface-controlled, with over 84% of the current showing capacitive behavior including both EDLC and pseudocapacitive adsorption of I_3_^–^ on the positively charged partially oxidized triindole backbone. While part of this extra capacity may come from the higher surface area of **TRIPh-d** (EDLC), a significant contribution from pseudocapacitance is also expected. It should be noted that the redox-active triindole framework can be partially oxidized during cycling, generating delocalized radical cations that electrostatically interact with the iodine and polyiodide species (I_3_^–^, I_5_^–^) suppressing the shuttle effect and improving iodine utilization. These sites also promote pseudocapacitive redox processes, leading to reversible charge storage and improved overall battery performance. The areal capacity at the same current density is approximately 0.09 and 0.14 mAh cm^–2^ for **TRIPh-m** and **TRIPh-d**, respectively. The superior charge storage capability of **TRIPh-d** was further evidenced by its higher energy density of ∼228 W h kg^–1^, compared with ∼157 W h kg^–1^ for **TRIPh-m**. Both **TRIPh-d** and **TRIPh-m** exhibit comparable power densities (≈1000 W kg^–1^), which aligns with their shared capacitive charge storage mechanism and a likely rate-limiting I^–^/I_3_^–^/I_2_ redox step. The rate performance of **TRIPh-d** at different current densities in the Zn/I_2_ battery system is shown in Figure 8c. **TRIPh-d** also exhibited excellent rate capability in the range of 0.5–2 A g^–1^. Within this region, both the specific capacities and Coulombic efficiencies remained nearly constant, with Coulombic efficiencies close to 100%, confirming the highly reversible I^–^/I_2_ conversion. At lower current densities, however, the system displayed reduced stability, which can likely be attributed to a greater contribution from parasitic side reactions.

**Figure 8:**
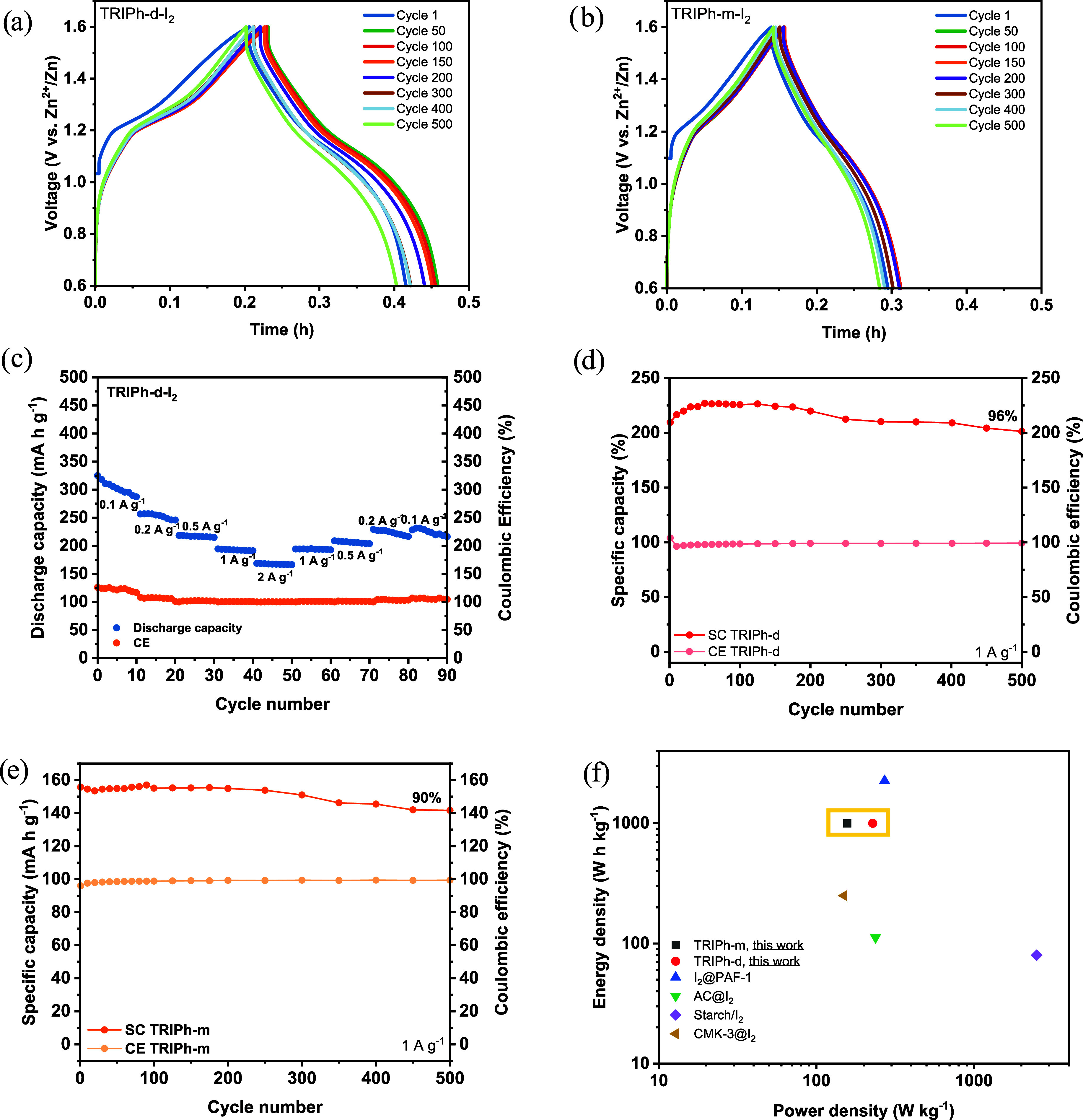
(a) GCD curves at 1 A g^–1^ in the potential range of 0.6–1.6 V of (a) **TRIPh-d** and (b) **TRIPh-m.** (c) Rate performance and Coulombic efficiencies at different current densities for **TRIPh-d**. d) Capacity retention and Coulombic efficiency during cycling at 1 A g^–1^ of **TRIPh-d** and (e) **TRIPh-m**. (f) Ragone Plot of different Zn–I_2_ battery systems using **TRIPh** networks and recently reported based on iodine capturing porous materials.

The cycling performance of **TRIPh**-based ZIBs at 1 A g^–1^ exhibited high capacity retention with Coulombic efficiencies approaching 99% for both polymer networks (Figure 8d,8e). **TRIPh-d** displayed comparable stability to **TRIPh-m**, retaining 96 and 90% of their initial capacities, respectively, after 500 cycles. Notably, **TRIPh-d** showed a gradual increase in capacity during the initial cycles before the expected long-term decrease. This behavior can be attributed to a residual activation process, likely arising from its higher number of accessible active sites for iodine storage compared to **TRIPh-m**. Long-term cycling was also achieved at 5 A g^–1^ with **TRIPh-d** based ZIB, showing a capacity retention of 72% after 10,000 cycles, maintaining a high Coulombic efficiency of ca. 100% (Figure S22). The higher cycling stability at elevated current densities further demonstrates the excellent rate performance of the **TRIPh-d** network in Zn/I_2_ battery systems. The small semicircle radius in the Nyquist plot obtained from electrochemical impedance spectroscopy (EIS) for the **TRIPh-d**–based Zn/I_2_ cell indicates its low charge-transfer resistance (Figure S23). After cycling, the enlarged semicircle radius suggests partial cell degradation. Notably, even after cell cycling, neither the electrolyte nor the separator exhibited coloration, pointing to a negligible iodine shuttle effect (Figure S24).

The Ragone plot in Figure 8f compares the performance of **TRIPh**-based Zn/I_2_ batteries with previously reported data for iodine-capturing porous organic and carbon-based materials. In particular, **TRIPh-d** demonstrates a favorable combination of energy and power densities, highlighting the advantage of its higher density of active sites compared to **TRIPh-m**. As summarized in Table S8, several studies have already demonstrated high capacity and cycling stability by introducing high-surface-area materials or active sites that minimize the shuttle effect through physical trapping or efficient adsorption of soluble polyiodides in aqueous Zn/I_2_ batteries. While these synthetic approaches often rely on high-energy processes or complex procedures, the polymer networks developed here can be synthesized under milder conditions and at lower cost, offering a promising foundation. Moreover, knitting polymers have been shown to provide suitable capabilities and robust cycling performance. Future work in this direction will focus on tailoring the redox mechanism by modifying the *N*–Ph pendant group to further improve the performance of these **TRIPh-d** based Zn–I_2_ batteries.

## 4 Conclusions

New thermally stable porous polymers have been synthesized through AlCl_3_-mediated Friedel Crafts reactions of triphenyltriindole by two different strategies: by thermal activation in CH_2_Cl_2_ solution (**TRIPh-d**) and by mechanosynthesis (**TRIPh-m**). I_2_ adsorption capacities of these polymers were investigated. Characterization of the polymer synthesized by the two routes evidenced similar chemical structures, although significant differences in the resulting porosity were found. In spite of the different surface areas (520 and 54 m^2^g^–1^) both materials show remarkable iodine adsorption capacity in solution, reaching values of 1817 and 1530 mg g^–1^. This performance is attributed to their persistent porosity and the ease with which the constituting triphenyltriindole units undergo oxidation in the presence of iodine, forming radical cations that enhance iodine adsorption via electrostatic interaction.

Comparison with a truxene-based analog **TX-m**, which has a much higher surface area (1025 m^2^·g^–1^) but lacks nitrogen sites, confirms that redox-active nitrogenated positions play a more critical role than porosity alone. **TX-m** shows faster iodine diffusion in vapor due to its high surface area, but its adsorption capacity is lower. These differences were more pronounced in solution than in vapor, suggesting that the oxidation-driven adsorption process is favored in solution.

In addition to iodine capture, **TRIPh** polymers demonstrate excellent filtration capabilities, efficiently purifying 30 mL of a 300 mg·L^–1^ iodine solution within just 2 h. Their high adsorption capacity and environmentally friendly synthesis make them promising candidates for iodine purification processes. The ability of **TRIPh** networks to stably incorporate iodine/polyiodide species within its polymer network makes them attractive as cathode material in metal/I_2_ batteries. **TRIPh-d**, with its larger number of accessible *N* active sites compared to **TRIPh-m**, proved to be a promising cathode material for Zn/I_2_ batteries delivering a high specific capacity of 228 mA h g^–1^ at 1 A·g^–1^, maintaining 99% Coulombic efficiency, and achieving 72% capacity retention after 10,000 cycles at 5 A g^–1^, demonstrating its potential for durable and high-performance energy storage applications. Overall, this work highlights how tailoring monomer selection and synthesis conditions enables the design of multifunctional polymer networks that unite environmental remediation with sustainable energy storage, showcasing their potential as transformative materials.

## Supplementary Material



## Notes

Details of the experimental protocols used for monomer synthesis, supplementary data on material characterization before and after adsorption, comprehensive descriptions of the adsorption models evaluated, kinetic analyses of both adsorption and photocatalytic processes, and a comparative assessment of the adsorption performance of the synthesized polymers relative to other known adsorbents (PDF)
