# Supplementary material for: Dual-Functional Triphenyltriindole “Knitting Polymers” for Efficient Iodine Capture and Zn–I2 Batteries
Source: ACS Appl Eng Mater. 2025 Nov 13;3(11):4074–85. doi: 10.1021/acsaenm.5c00709 (PMC12670518; doi:10.1021/acsaenm.5c00709)
Supplement: Supplementary file 1 [file em5c00709_si_001.pdf]

Supporting Information

Dual-functional Triphenyltriindole "knitting polymers" for Efficient Iodine Capture and Zn-I_2 Batteries

Nayara Méndez-Gil[a,b,†] Paula García-Balaguer[a,b,†], Lidia Martínez[a], Yves Huttel[a], Mohammad Afsar Uddin[a], María Luisa Ferrer[a,] and Berta Gómez-Lor[a,*]*

[a]Instituto de Ciencia de Materiales de Madrid (ICMM), CSIC, C/ Sor Juana Inés de la Cruz 3, Madrid 28049, Spain.

[b] Universidad Autónoma de Madrid, 28049 Madrid, Spain.

†These authors contributed equally.

*Corresponding author: bgl@icmm.csic.es and mferrer@icmm.csic.es

Table of contents

1. Methods and materials S3

2. Synthesis of monomers (**TxMe** and **TPh**) and the soluble **TRIPh** model S5

3. Characterization of **TRIPh-d**, **TRIPh-m** and **TX-m** polymers.......................................S8

4. Capture and release of Iodine vapor using **TRIPh-d**, **TRIPh-m** and **TX-m** polymers..S12

4.1 Iodine vapor adsorption S12

4.2 Iodine vapor desorption S16

5. Iodine capture in hexane using **TRIPh-d**, **TRIPh-m**, and **TX-m** polymers S18

5.1 Langmuir isotherm model..S21

5.2 Freundlich isotherm model S22

5.3 Adsorption kinetic study of iodine in hexane by **TRIPh-d**. S24

6. Adsorption of Iodine from aqueous media by **TRIPh-d**……………………………….S30

7. Characterization of iodine-loaded polymers.. ....S30

8. Electrochemical measurements S32

8.1 Preparation Iodine loaded polymer for electrochemical measurements S32

8.2 Evaluation of iodine-loaded TRIPh-d as cathode in Zn/I_2 batteries……………………S26

9. References S41

1. Methods and materials

Thermogravimetric analyses (TGA) were performed on a TA TGA Q-500 instrument using 5 mg of polymer. Samples were heated at 10 °C/min starting from room temperature to 800 °C under an air flow rate of 40 mL/min. Elemental analyses (%C, %N and %H) were determined in a Carlo-Erba EA 1108 analyzer. Fourier Transform Infrared Spectra (FT-IR) were recorded on a Perkin-Elmer RX-1 instrument with a resolution of 1.0 ± 0.1 cm^{-1}. Nuclear magnetic resonance (NMR) spectra of liquid samples were recorded with a Bruker-300 Ultra Shield (300MHz for ^{1}H and 75MHz for ^{13}C). Solid-state ^{13}C MAS-NMR measurements were recorded with a Bruker AV400 WB spectrometer (400 and 100 MHz Larmor frequencies, using 4 mm MAS probes spinning at 10 kHz). Scanning electron microscopy (SEM) images were obtained with a FEI Nova NANOSEM 230 FE-SEM microscope. Measurements were carried out directly on the dispersed powder on a double-sided adhesive surface operating at 0.5 kV. Porosity analysis and specific surface areas, were performed from N_2 adsorption/desorption isotherms measured at 77 K on a Micromeritics ASAP 2020 Plus Adsorption Analyzer using the Brunauer-Emmett-Teller (BET) theory for surface area calculation. Prior to measurement, the samples were degassed overnight at 200 ºC under vacuum. Pore size distribution was determined using NLDFT models assuming predominant slit pores using QuadraWin software for data reduction). The samples were then transferred from atmosphere to the X-ray Photoemission Spectroscopy (XPS) chamber with a base pressure of 10^{-10} mbar through a load-lock fast entry chamber. The XPS chamber is equipped with a hemispherical electron energy Analyzer (SPECS Phoibos 100 spectrometer) and an AlKα (1486.29 eV) X-ray source operated at 150 W. The angle between the hemispherical analyzer and the plane of the surface was kept at 60°. I 3d core level spectra was recorded using an energy step of 0.1 eV and a pass-energy of 20 eV. Data processing was performed with CasaXPS software (Casa software Ltd, Cheshire, UK). The contributions of the AlKα satellite lines were subtracted. The absolute binding

energies (BE) of the photoelectron spectra were determined by referencing to the C1s at 285 eV. [1] UV-Visible and diffuse reflectance spectra were achieved with a Shimadzu UV-2401 PC spectrophotometer. Renishaw Ramascope 2000. Raman, Laser wavelength: 514.5 nm (Ar-ion laser). Normal (non-confocal) mode: Slit width: 50 μm. CCD: 206–225 pixels. Cyclic voltammetry (CV) tests were made in an AutoLab potentiostat. The potential sweep rate from 0 to 0.6 and 1.4 V vs Ag/AgCl was fixed to 0.5 mV s^{-1}. Galvanostatic charge and discharge (GCD) measurements were performed in a VMP-3e Biologic electrochemical working station. All cells were preconditioned at 0.6-1.6 V performing consecutive CVs at different scan rates. The cycling life of the batteries was tested by GCD cycling at 0.1 A g-1, using a LBT21084 battery test system (Arbin Instruments). Electrochemical experiments were carried out at 20°C.

All reagents required for the synthesis of the monomers, and the iodine were purchased from Sigma-Aldrich and used as received. Solvents were purchased from Fischer Sci. and used without further purification

2. Synthesis of monomers (TxMe and TPh) and the soluble TRIPh model.

The synthesis of the triphenyltriindole **TPh**[2] and hexamethyltruxene **TxMe** [3, 4] was performed following the procedures shown in Scheme 1 and 2 respectively, previously described in the literature.

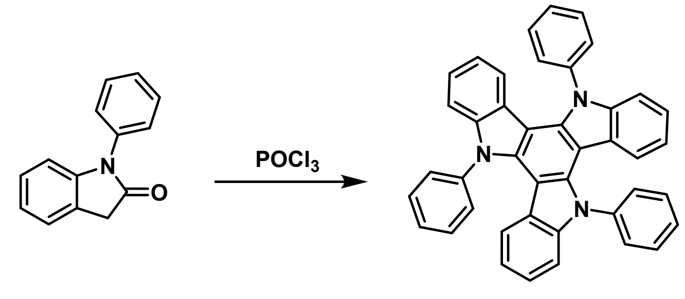

TPh

Scheme S1. Synthetic route to **TPh** monomer

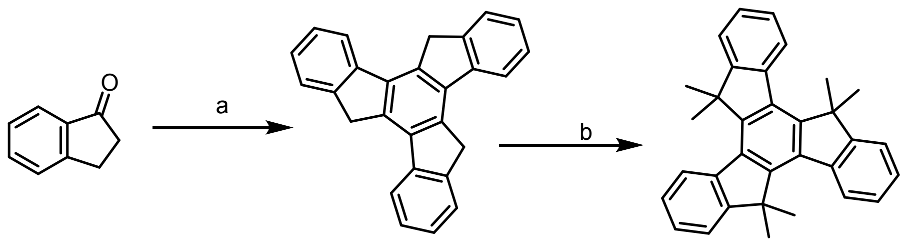

TxMe

(a) p-Toluene sulfonic acid, propionic acid; 1,4-dichlorobenzene; (b) tBuOK, MeI, DMSO

Scheme S2. Synthetic route to **TxMe** monomer.

Synthesis of the soluble TRIPh model: A mixture of TPh (100 mg, 0.17 mmol), Nonanoyl Chloride (0.13 mL, 0.7 mmol) and $AlCl_3$ (93 mg, 0.7 mmol) in dry DCM (2 mL) was stirred at room temperature for 2 hours in nitrogen atmosphere. Then the mixture was poured into ice and extracted with dichloromethane. After removing the solvent, the solid obtained was purified by column chromatography on silica gel, using a mixture of dichloromethane and heptane (8:1). The compound was obtained as a yellow solid (104 mg, 60% yield) ^{1}H NMR (300 MHz, $CDCl_3$) δ 7.91 (dd, J = 8.8, 1.6 Hz, 1H), 7.71 (d, J = 4.3 Hz, 3H), 7.67 – 7.60 (m, 2H), 7.43 (d, J = 8.7 Hz, 1H), 7.10 (d, J = 1.6 Hz, 1H), 2.62 (t, J = 7.4 Hz, 2H), 1.65 (m, J = 8.8 Hz, 2H), 1.44 – 1.21

(m, 11 H), 0.89 (m, J = 6.5 Hz, 2H). ^{13}C NMR (75 MHz, $CDCl_3$) δ 200.46, 144.49, 140.09, 138.16, 130.68, 130.62, 129.28, 128.11, 124.45, 122.06, 110.26, 105.64, 38.99, 32.07, 32.00, 29.80, 29.68, 29.43, 24.79, 22.85, 14.28. (75 MHz, CDCl3) δ. MALDI-TOF MS m/z 993.6 (M+); HRMS (MALDI-TOF) calcd for $C_{69}H_{75}N_3O_3$: 993.5803, found: 993.5780. Elemental Analysis (%) Calculated: C 84.34, H 7.60, N 4.23, O 4.83. Experimental: C 84.75, H 7.44, N 4.46.

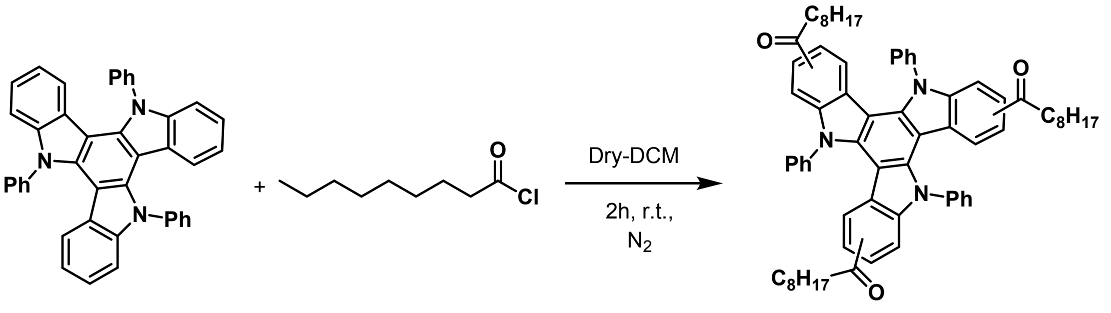

TPh

TPh-nonanoyl

Scheme S3. Synthetic route to the soluble **TRIPh** model.

^1H NMR spectrum of **TRIPh- model** in $CDCl_3$

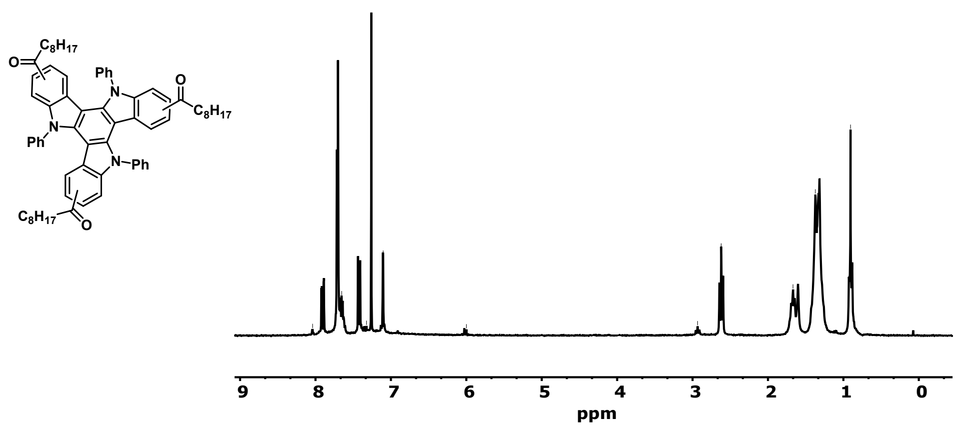

^{13}C NMR spectrum of **TRIPh- model** in $CDCl_3$

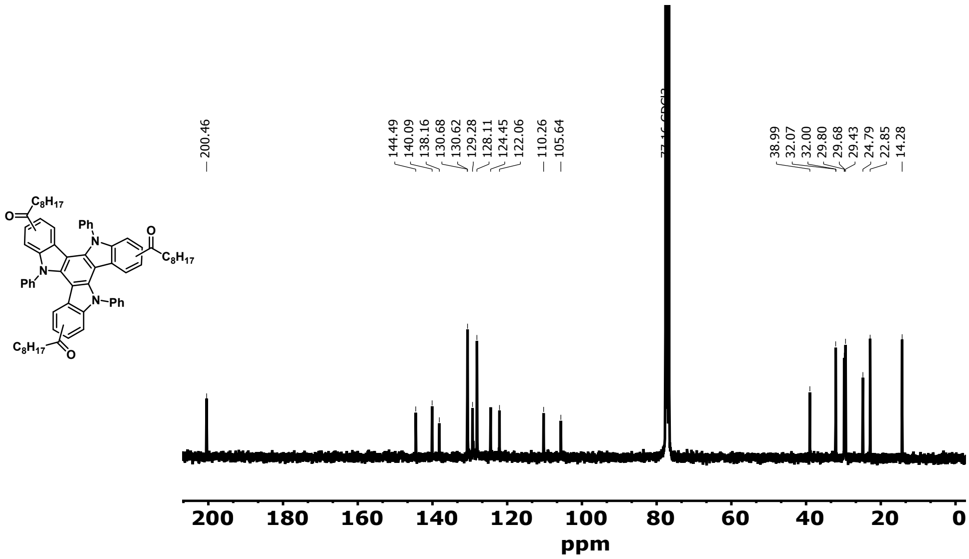

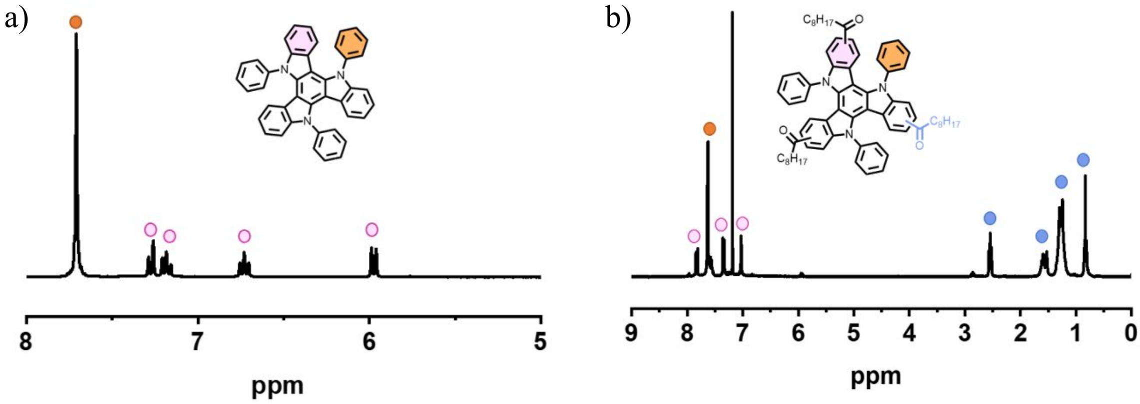

Figure S1. ^{1}H-NMR (300 MHz, $CDCl_3$) of (a) TPh monomer and (b) the nonanoyl derivative, showing that the signals affected upon reaction are only those corresponding to the triindole platform.

3. Characterization of TRIPh-d, TRIPh-m and TX-m polymer

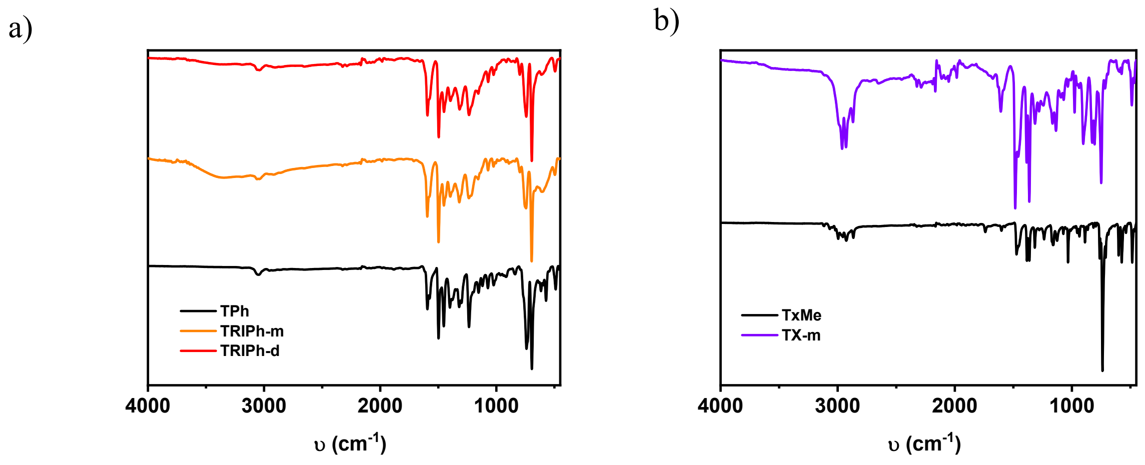

Figure S2. Stacked FTIR spectra of (a) **TPh, TRIPh-m, TRIPh-d**; (b) **TX-m** and **TxMe**.

Table S1. Elemental analysis of POPs.

Polymer	Yield (%)	Data	%C	%H	%N	C/N
**TRIPh-d***	56	Calc.	87.94	5.14	6.91	15
		Exp.	79.05	4.68	5.68	16
**TRIPh-m***	62	Calc.	87.94	5.14	6.91	15
		Exp.	79.32	4.21	5.91	16
**TX-m***	93	Calc.	93.2	6.80	------	------
		Exp.	84.01	5.84	------	------

*The theoretical elemental composition was calculated from the repeating unit of the triphenyltriindole or truxene monomer and 1.5 methylene linkers per unit, accounting for the shared nature of each methylene bridge between two monomers.

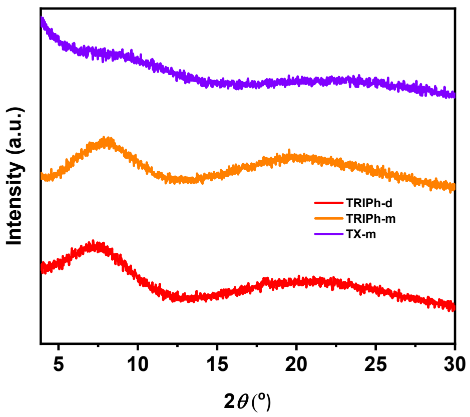

Figure S3. Powder X-ray diffraction of **TRIPh-d** (red)**, TRIPh-m** (orange) and **TX-m** (purple).

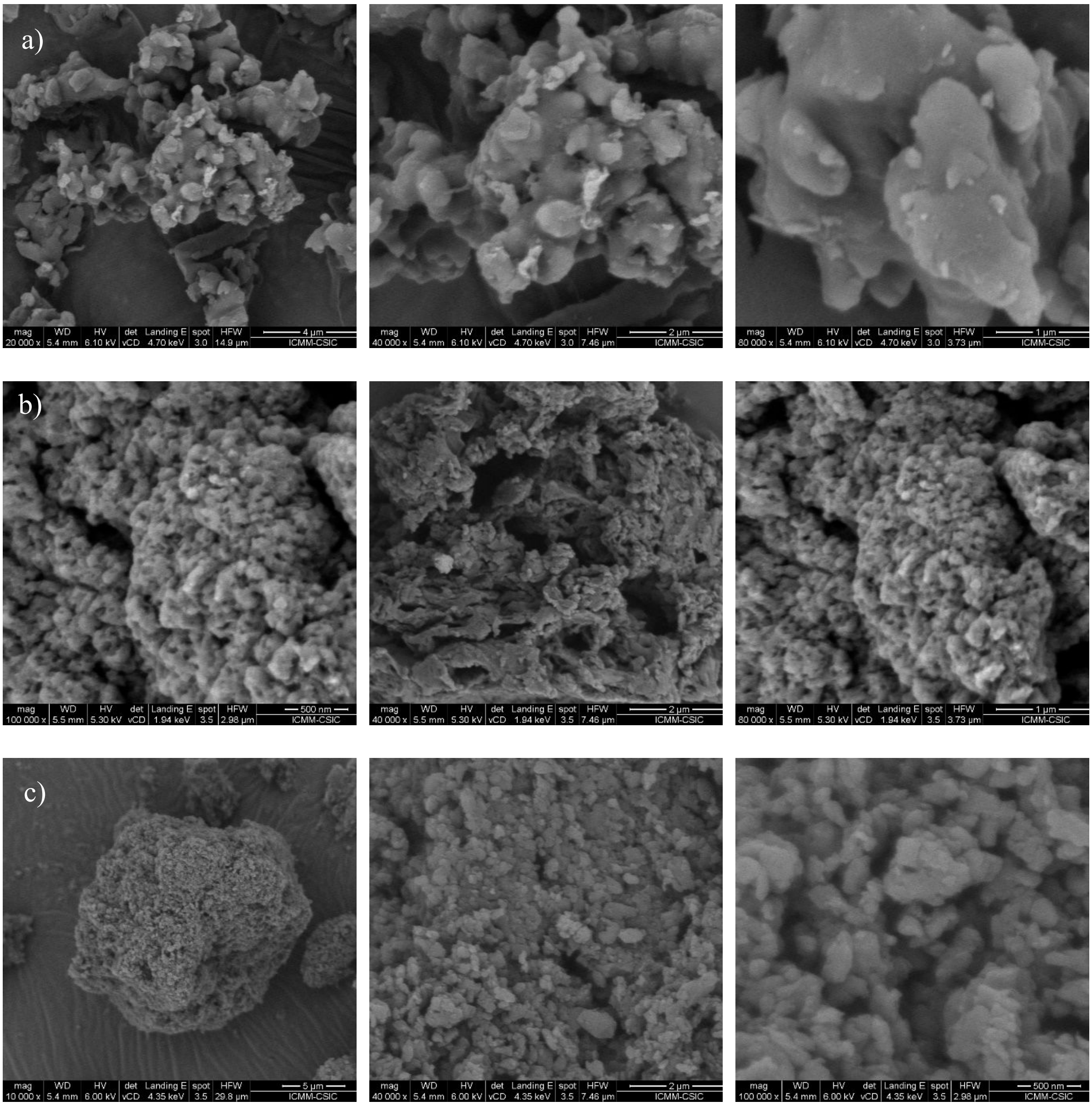

Figure S4. SEM images at different magnifications of (a) **TRIPh-d**, (b) **TRIPh-m** and (c) **TX-m.**

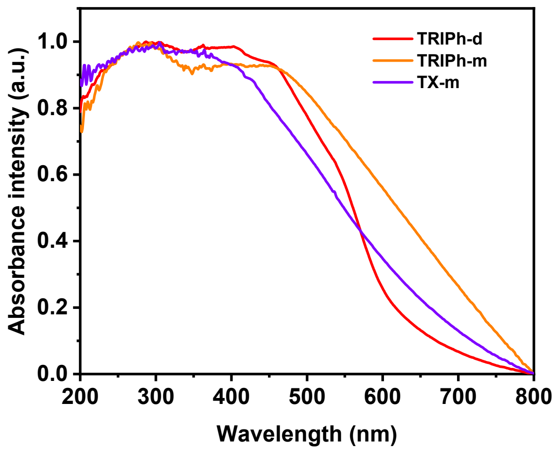

Figure S5. UV-vis solid absorption of **TRIPh-d** (red), **TRIPh-m** (orange), **TX-m** (purple).

4. Capture and release of Iodine vapor with TRIPh-d, TRIPh-m and TX-m polymers

4.1 Iodine vapor adsorption

25 mg of each polymer (**TRIPh-d**, **TRIPh-m** and **TX-m**) was introduced into individual vials saturated with nonradioactive iodine vapor at a temperature of 75 ºC and atmospheric pressure. Subsequently, a gravimetric method was employed to assess the iodine uptake of the polymers at different time intervals. The iodine uptake was determined using the following formula

$Q_m = (m_2 - m_1)/m_1 \cdot 100\ wt\%$ Equation S1

Where Qm is the iodine uptake, m1 and m2 are the quantity of mass before and after iodine uptake in %.

The iodine uptake capacity of **TRIPh-d** was evaluated under various conditions. As shown in Figure S6, the maximum uptake (qm) under standard conditions (25 mg of polymer at 75 °C) was 282 wt%. Increasing the amount of polymer to 50 mg did not significantly affect the uptake capacity, which remained comparable at 264 wt%. In contrast, elevating the temperature from 75 °C to 100 °C resulted in a higher qm value of 350 wt%, indicating that increased temperature enhances adsorption performance. Nevertheless, to maintain consistency and enable direct comparison with previously reported studies, all adsorption experiments were conducted at 75 °C.

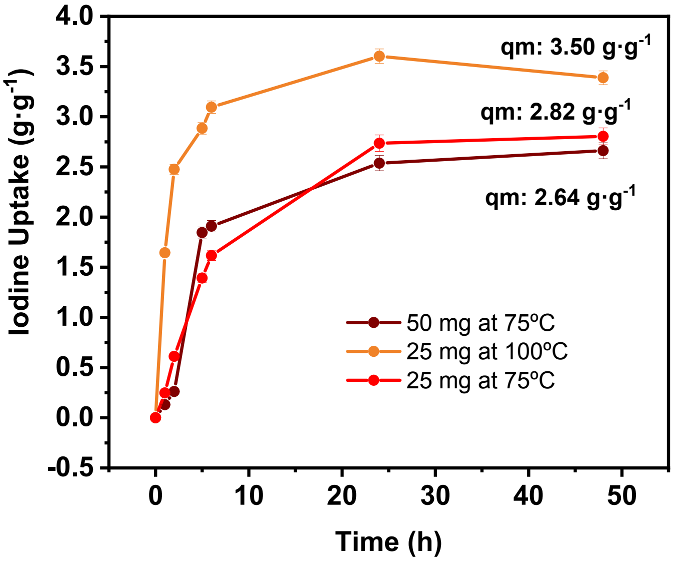

Figure S6. Vapor iodine uptake capacity of **TRIPh-d** under different experimental conditions.

Table S2. Comparison of the adsorption capacities of various adsorbents towards the studied iodine with that of **POPs** in vapor.

Adsorbent	BET surface (m^2g^{-1})	qm (%wt)	Temperature (°C)	Ref
**NR_POP-C2**	64	716	75	[5]
**AZO-POP-P**	127	356	75	[6]
**PTZ-TPC-MA**	132	198	75	[7]
**Ac_POP-5**	190	811	75	[8]
**H-C-CTPs**	640	460	75	[9]
**Car-CMP-3**	450	510	75	[10]
**BHCP-3**	810	619	75	[11]
**CzBMI-POP**	57	215	75	[12]
**P-AM**	2	277	85	[13]
**PAF-TDBA**	861	313	75	[14]
**PAF-DA**	684	226	75	[14]
**LNU-14**	344	223	75	[15]
**DADP-COF**	1140	420	75	[16]
**TAPT-COF-AB**	455	505	75	[17]
**iCOFs-AB-2**	1013	426	77	[18]
**KOH-AC**	1973	376	77	[19]
**PDA@PDVB1**	1551	217	75	[20]
**20CuS/ AC**	9	49	75	[21]
**TRIPh-d**	521	282	75	This work
**TRIPh-m**	54	257	75	This work
**TX-m**	1025	202	75	This work

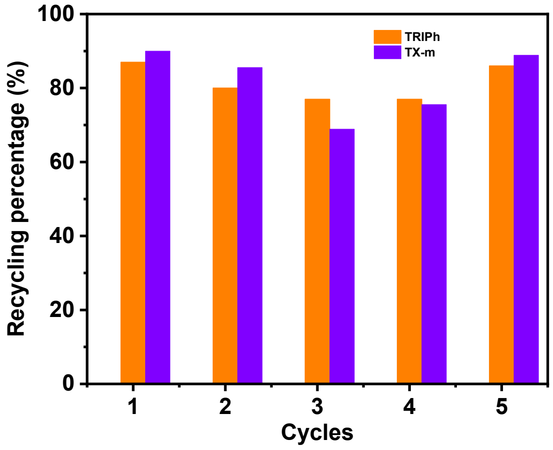

Figure S7. Adsorption performance of **TRIPh** (orange) and **TX** (purple)after subsequent adsorption-desorption cycles.

4.2. Iodine vapor desorption

5 mg of iodine-loaded **TRIPh-d**, **TRIPh-m** and **TX-m** were immersed in 15 mL of ethanol at room temperature and without stir. UV-Vis spectra were registered at various time intervals with a 0.2 cm cuvette. (Figure S7).

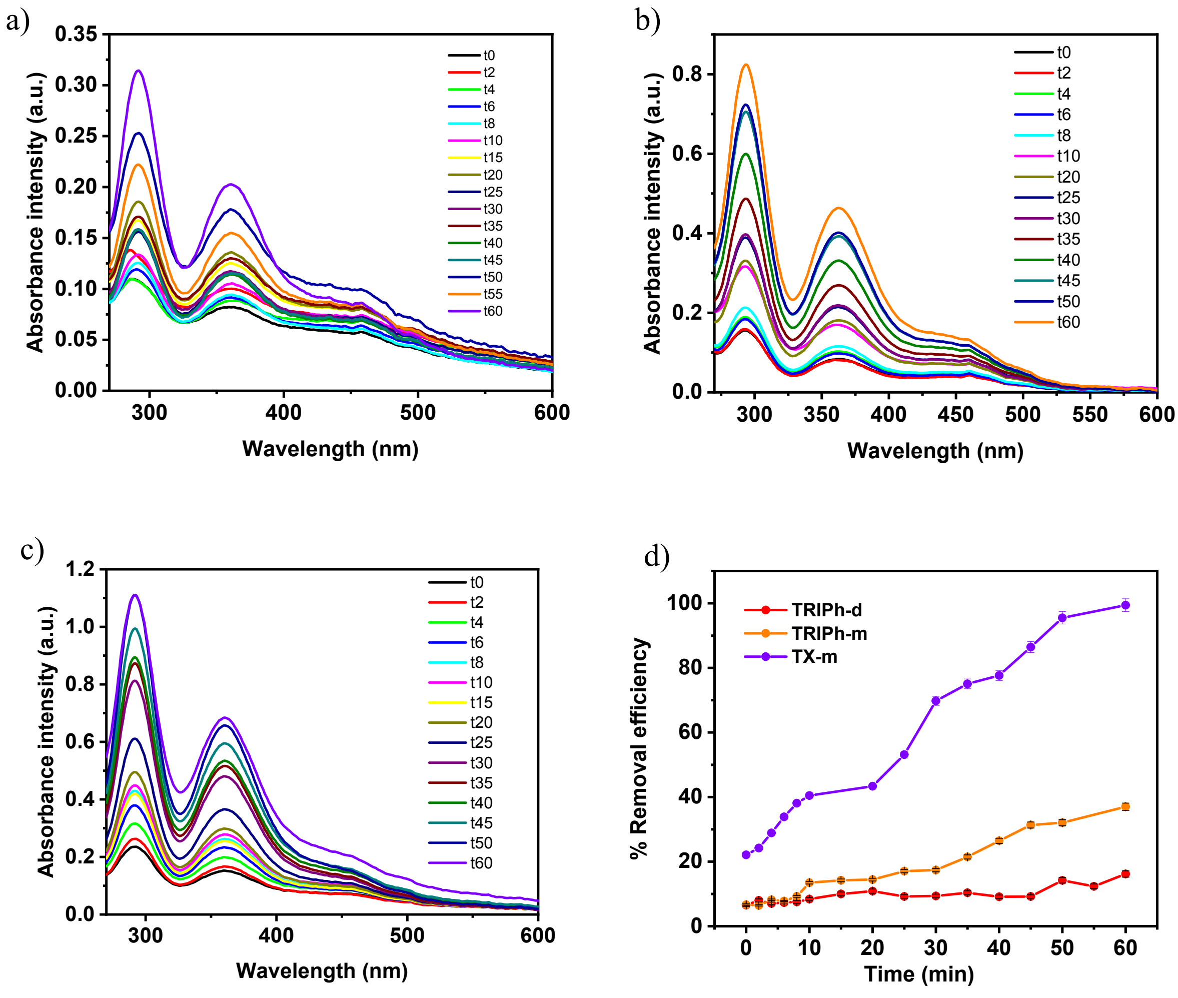

Figure S8. Desorption of absorbed iodine vapor from iodine-loaded (a) **TRIPh-d**, (b) **TRIPh-m** and (c) **TX-m** in ethanol and d) removal efficiency estimated error < 5%.

The variation of the adsorption maxima of iodine in the presence of **TRIPh-d, TRIPh-m** and **TX-m** with time, was analyzed by applying the pseudo-first order and pseudo-second order models. 25 mg of each polymer was measured by gavimetric method every 1 hour.

The pseudo-first-order kinetic model applies the equation:

$$\boldsymbol{ln\ (qe - qt) = ln\ qe - k_1 t} \qquad \text{Equation S2}$$

where q_e is the quantity of solute adsorbed per unit mass of the adsorbent at equilibrium, q_t is the quantity of solute adsorbed per unit mass of the adsorbent at time t, k_1 is the first-order kinetic constant and t is time.

The pseudo-second-order kinetic model applies the equation:

$$\boldsymbol{\frac{t}{qt} = \frac{t}{qe} - \frac{1}{k_2 q_e^2}} \qquad \text{Equation S3}$$

where q_e is the quantity of solute adsorbed per unit mass of the adsorbent at equilibrium, q_t is the quantity of solute adsorbed per unit mass of the adsorbent at time t, k_2 is the second-order kinetic constant and t is time.

Table S3. Calculated parameters of isotherm models for the adsorption of iodine in vapor onto POPs.

	Pseudo-first order				Pseudo-second order
	K_1 (min^{-1})	qe ($g\ g^{-1}$)	q_{exp}* ($g\ g^{-1}$)	R^2	R^2
**TRIPh-d**	0.137	2.59	2.82	0.976	0.368
**TRIPh-m**	0.128	2.46	2.57	0.998	0.991
**TX-m**	0.175	1.90	2.02	0.987	0.983

* q_{exp} is calculate represent the qt vs time, When the curve stabilizes at its maximum adsorption, that point will be the value of the $q_{exp.}$

5. Capture of Iodine from hexane solution with TRIPh-d, TRIPh-m and TX-m polymers

To establish the adsorption isotherm curves, different initial iodine solutions with concentrations varying from 900 to 7000 mg. L^{-1} (for TRIPh-d), from 100 to 7000 mg. L^{-1} (for TRIPh-m) and from 100-1500 mg. L^{-1} (for TX-m) were prepared.

5 mg of each polymer was added to 5 mL of each iodine solution and the mixture was placed in the dark without stirring for 24 h. UV-Vis spectra were recorded on 0.1 cm cuvettes for **TRIPh-d** and on 0.2 cm cuvettes for **TRIPh-m** and **TX-m**.

The amount adsorbed per unit mass of the solid at equilibrium was determined by the following relation:

$$Q_e = (C_i - C_e)/M \cdot V \qquad \text{Equation S4}$$

where Qe is the amount of the iodine adsorbed by the porous polymer (mg. g^{-1}), C_i and C_e the initial and the equilibrium concentrations of the iodine solution, M the weight of adsorbent (g) and V the working volume on liter (L).

The adsorption isotherm curves were obtained by plotting the quantity adsorbed per unit mass of solid Qe (mg /g) as a function of the concentration of the adsorbent in equilibrium Ce (mg /L) in the solution at room temperature.

Table S4. Comparison of the adsorption capacities of various adsorbents towards the studied iodine with that of **POPs** in organic solvent.

Adsorbent	BET surface (m^2g^{-1})	qm (mg/g)	Conditions		Ref
			Polymer/solvent (g/mL)	[C] (mg/L)/solvent	
**BHCP-3**	810	54[a]	2	50-100/cyclohexane	11
**TAPDA-HCCP**	6	1150[a]	1	200-1000/ cyclohexane	22
**PT-POP**	475	345[a]	6 mg	200600/cyclohexane	23
**POP-PDTB**	5	1276[b]	1	n-hexane	24
**POP-PDTE**	16	1650[b]	1	n-hexane	24
**CYTR-PP**	36	1582[a]	10 mL	100-3000/n-hexane	25
**PPPy**	9	813[a]	0.5	200-500/cyclohexane	26
**iPOP-Bpy**	364	1010[a]	0.5	n-hexane	27
**CTAPA**	8	934[a]	0.2	50-300/n-hexane	29
**TpPa-TDI-AEP**	34	1502[b]	0.2	300-500/n-hexane	30
**C6-TRZ-TPA COF**	1058	238[a]	1	50-2000/cyclohexane	31
**KOH-AC**	1973	460[a]	1	cyclohexane	19
**K-RPC**	602	411[a]	1	cyclohexane	32
**TRIPh-d**	521	1817[b]	1	900-7000/ n-hexane	This work
**TRIPh-m**	54	1530[b]	1	100-7000/n-hexane	This work
**TX-m**	1025	153[b]	1	100-1500/n-hexane	This work

[a]qm calculated thought fitting Langmuir isotherm, [b]qm calculated through the stabilization of the curve representing qe *vs* ce

5.1 Langmuir isotherm model

The Langmuir isotherm is a simple model of monolayer adsorption. It is the most used model to comment the results found during the adsorption of organic compounds in Iodine in hexane solution. It is described by the following relation:

$$\mathbf{Ce/Qe = 1/\ KL \cdot q_m + Ce/q_m} \quad \text{Equation S5}$$

Q_e: Amount of substance adsorbed per unit mass of the adsorbent at equilibrium (mg/g).

K_L: Constant corresponding to the adsorption energy.

q_m: Maximum adsorption capacity (mg/g).

C_e: Equilibrium concentration of the adsorbate (mg/L).

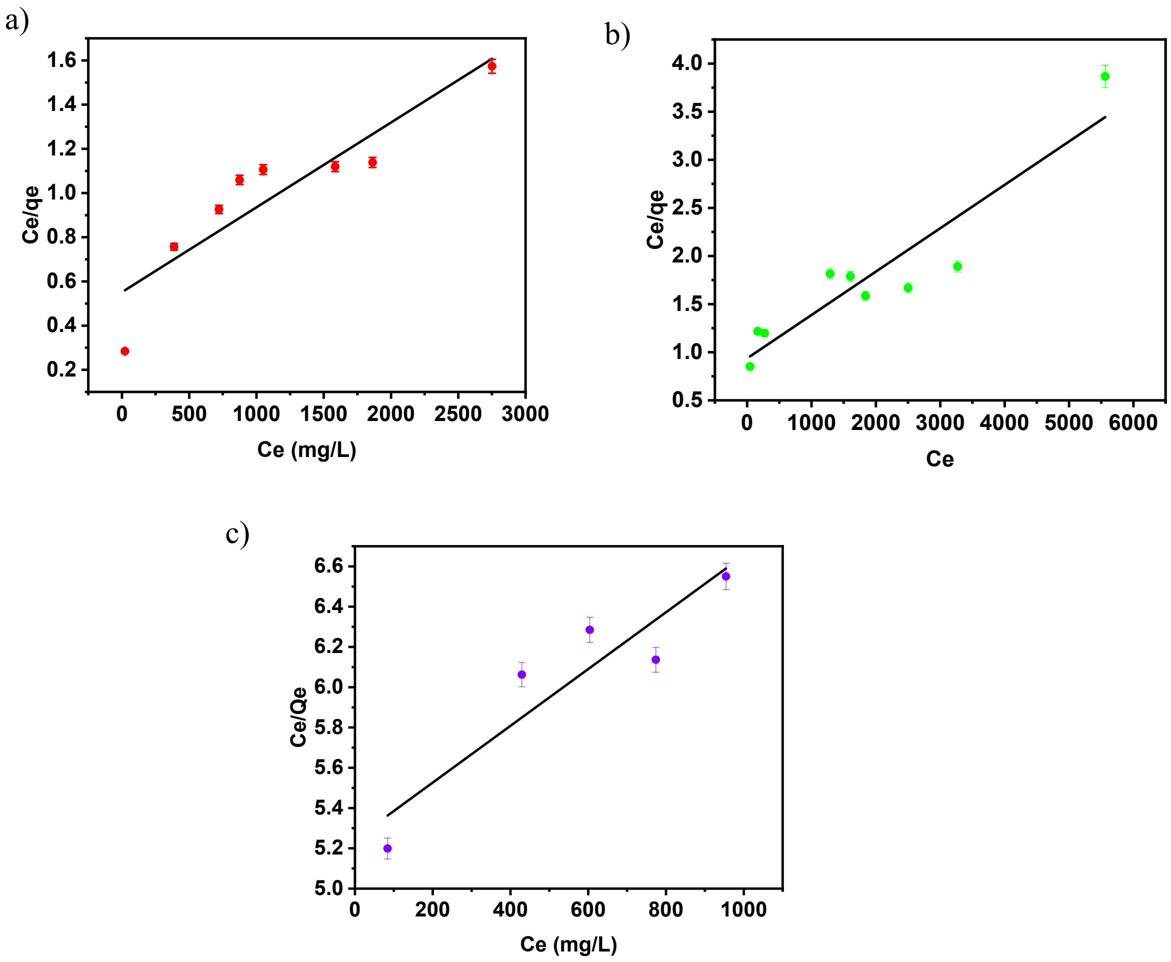

Figure S9. Langmuir isotherm model for Iodine in hexane solution onto (a) **TRITPh-d**, (b) **TRIPh-m** and (c) **TX-m** estimated error < 5%.

5.2 Freundlich isotherm model

The Freundlich isotherm is an empirical equation widely used for the practical representation of adsorption equilibrium.

It is presented in the form:

$$\textbf{Log Qe = 1/n Log Ce + Log Kf} \quad \text{Equation S6}$$

Q_e: quantity of solute adsorbed per unit mass of the adsorbent at equilibrium.

K_f: Freundlich constant associated with the adsorption capacity.

n: Freundlich energy parameter.

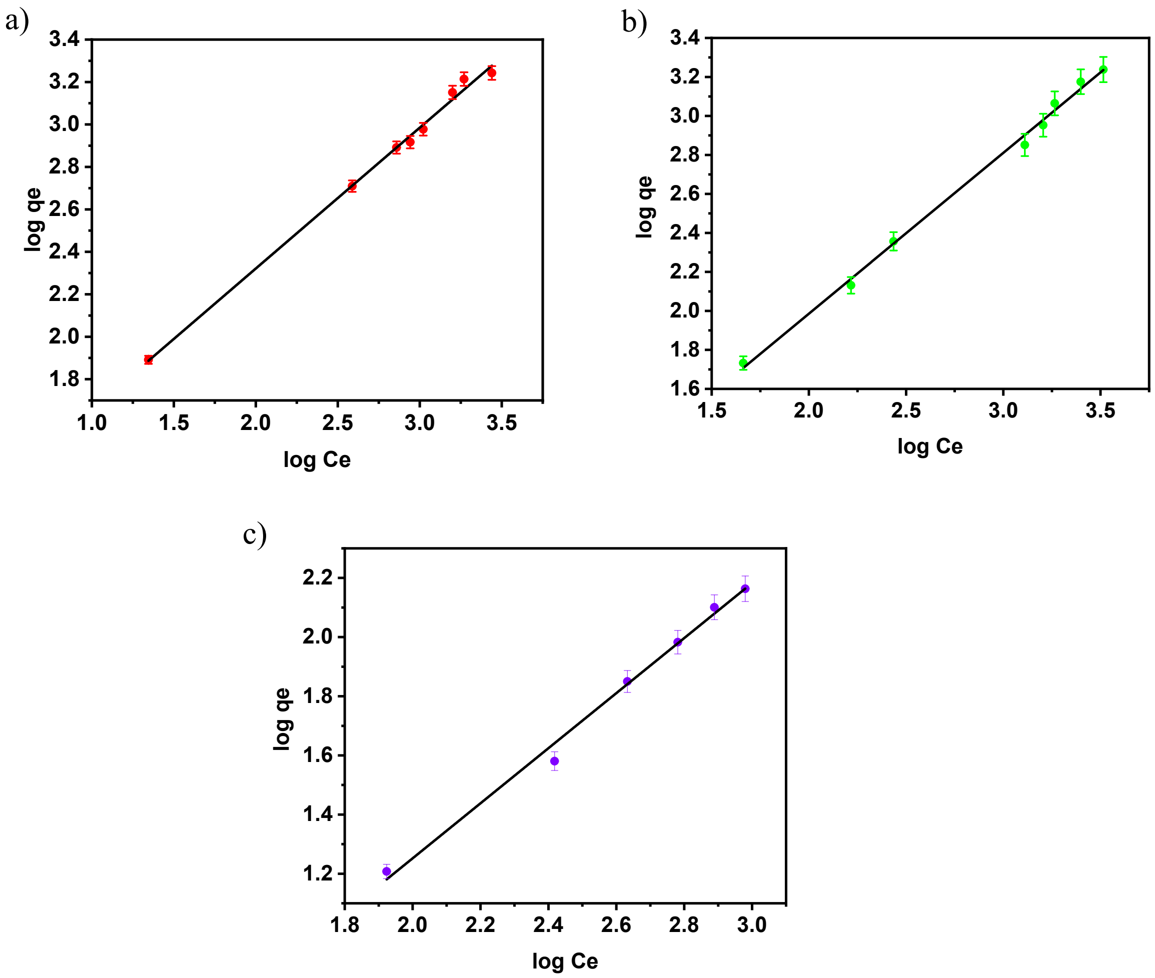

Figure S10. Freundlich isotherm model for Iodine in hexane solution onto (a) **TRIPh-d, (**b) **TRIPh-m** and (c) **TX-m** error estimate < 5%.

Table S5. Calculated parameters of isotherm models for the adsorption of iodine in n-hexane onto POPs.

**Polymer**	**Langmuir isotherm model**			**Freundlich isotherm model**		
	K_L (L mg^{-1})	q_m (mg. g^{-1})	R^2	K_f	n	R^2
**TRIPh-d**	$6.96 \cdot 10^{-4}$	2605	0.80356	9.87	1.50698	0.99428
**TRIPh-m**	$4.82 \cdot 10^{-4}$	2218	0.84274	23.7	1.92715	0.99624
**TX-m**	$3.88 \cdot 10^{-4}$	490.2	0.54562	0.245	1.07445	0.99057

5.3 Adsorption kinetic study of iodine in hexane by TRIPh-d.

The variation of the adsorption maxima of iodine in the presence of **TRIPh-d** with time, was analyzed by applying the pseudo-first order and pseudo-second order models. 1.5 mg of **TRIPh-d** in 2.5mL of a 300 mg/L iodine solution in hexane was measured by UV-Visible every 5 minutes for 1 hour with a 1cm cuvette.

The pseudo-first-order kinetic model applies the equation:

$$\boldsymbol{ln\,(qe - qt) = ln\,qe - k_1 t} \qquad \text{Equation S2}$$

where q_e is the quantity of solute adsorbed per unit mass of the adsorbent at equilibrium, q_t is the quantity of solute adsorbed per unit mass of the adsorbent at time t, k_1 is the first-order kinetic constant and t is time.

The pseudo-second-order kinetic model applies the equation:

$$\boldsymbol{\frac{t}{qt} = \frac{t}{qe} - \frac{1}{k_2 q_e^2}} \qquad \text{Equation S3}$$

where q_e is the quantity of solute adsorbed per unit mass of the adsorbent at equilibrium, q_t is the quantity of solute adsorbed per unit mass of the adsorbent at time t, k_2 is the second-order kinetic constant and t is time.

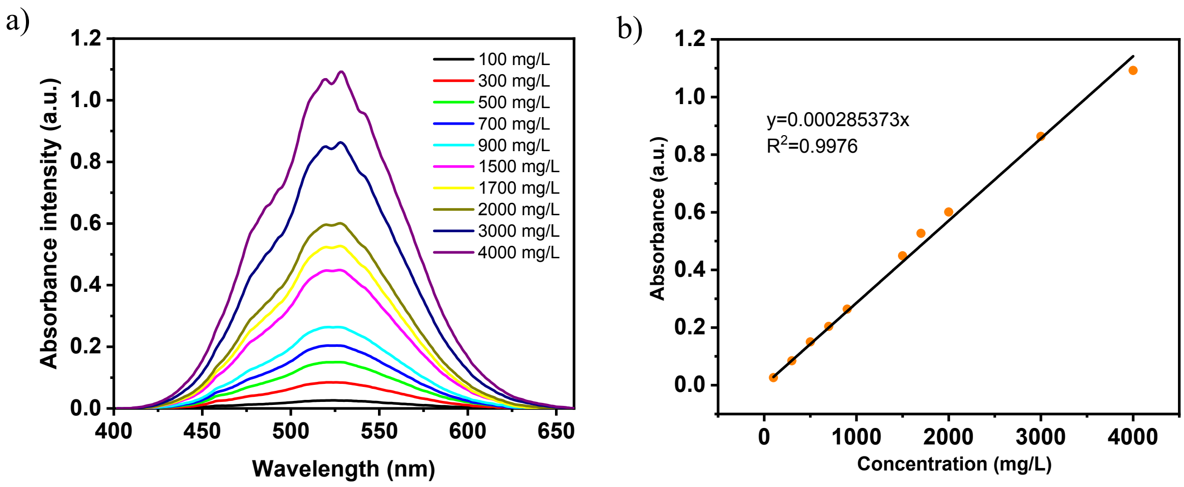

Figure S11. Calibration curve of iodine in hexane with a 0.1 cm cuvette (a) UV-Vis (b) $y=2.85\cdot10^{-4}x$

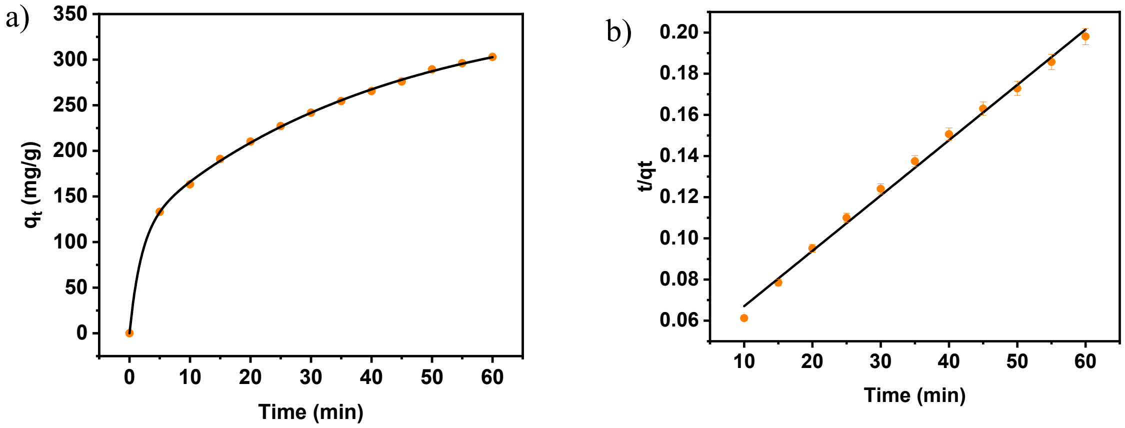

Figure S12. Qt vs time (a), pseudo-second order kinetic (b) in a 300mg/L of iodine in hexane of **TRIPh-d** estimated error < 5%**.**

Table S6. The data adsorption kinetics of **TRIPh-d** towards 300mg/L solution of iodine in hexane.

	**K_1 (min^{-1})**	**qe (mg g^{-1})**	**q_{exp} (mg g^{-1})**	**R^2**
**Pseudo-first order**	0.0578	286	312	0.927
**Pseudo-second order**	$1.80 \cdot 10^{-4}$	372	312	0.994

6. Adsorption of Iodine from aqueous media by TRIPh-d

To establish the adsorption isotherm curves, different initial iodine solutions with concentrations varying from 500 to 2538 mg. L^{-1} (for **TRIPh-d**). 5 mg of **TRIPh-d** was added to 5 mL of each iodine solution and the mixture was placed in the dark without stirring for 24 h. A 20 µL aliquot of the solution was diluted in 1 mL of water and the UV-Vis spectra were recorded on 0.2 cm cuvettes. The Langmuir and Freundlich isotherm were measured using equation S4-S6. In this case, the correlation coefficient obtained for the Langmuir isotherm model (R^2 = 0.9995) was higher than that of the Freundlich model (R^2 = 0.9149), suggesting that iodine adsorption from aqueous solution occurs as a monolayer and is homogeneously distributed on the surface of the polymer.

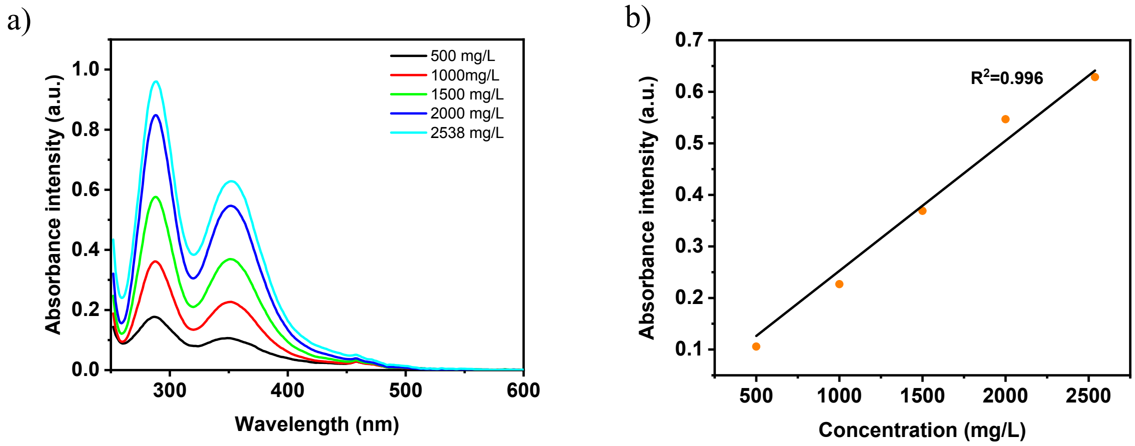

Figure S13. Calibration curve of iodine in hexane with a 0.1 cm cuvette (a) UV-Vis (b) $y=2.53\cdot10^{-4}x$.

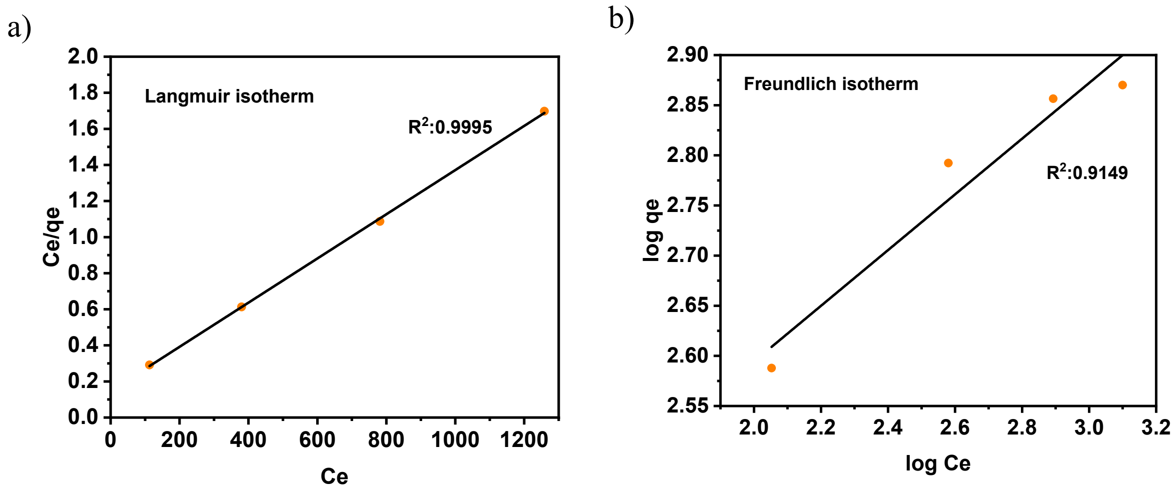

Figure S14. (a) Langmuir (y= 0.00122x+ 0.14699, R^2: 0.9995) and (b) Freundlich (y= 0.2778x+ 2.03878, R^2: 0.91489) isotherm model for aqueous iodine solution onto **TRIPh-d**.

Table S5. Calculated parameters of isotherm models for the adsorption of iodine in water onto **TRIPh-d**.

Polymer	Langmuir isotherm model			Freundlich isotherm model		
	K_L (L mg^{-1})	q_m (mg. g^{-1})	R^2	K_f	n	R^2
**TRIPh-d**	$8.3\cdot10^{-3}$	819	0.9995	109	3.5997	0.9148

To evaluate the robustness of the adsorption process, the iodine uptake of TRIPh-d was measured in aqueous solution in the presence of different competing ions. These experiments were conducted by monitoring the change in iodine concentration in 5 mL of a 1000 mg/L aqueous iodine solution with 5 mg of polymer. Selectivity was assessed by adding interfering ions (Cl^-, NO_3^-, and CO_3^{2-}) to reach a final concentration of 0.125 mol/L. Despite the presence of these ions, the adsorption efficiency remained approximately 56%, indicating high selectivity of the material toward iodine under the tested conditions.

A similar experiment was performed to evaluate the effect of pH on adsorption capacity. As shown in Figure S15, only slight variations in iodine uptake were observed across different pH values

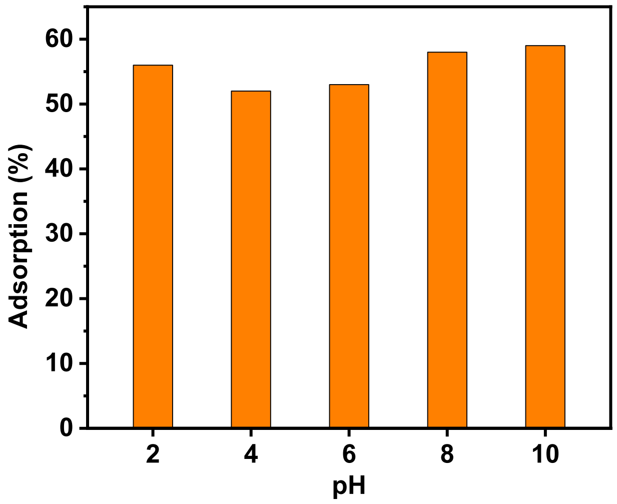

Figure S15. The iodine adsorption capacity of **TRIPh-d** in aqueous iodine solution under different pH conditions.

7. Characterization of iodine-loaded polymers.

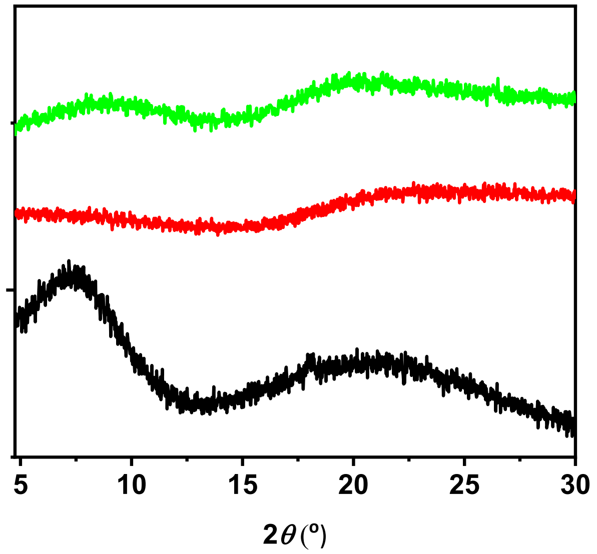

Figure S16. Powder X-ray diffractograms of **TRIPh-d** Comparison of the bare polymers (black) and iodine-loaded in vapor (red) and iodine adsorption in hexane (green).

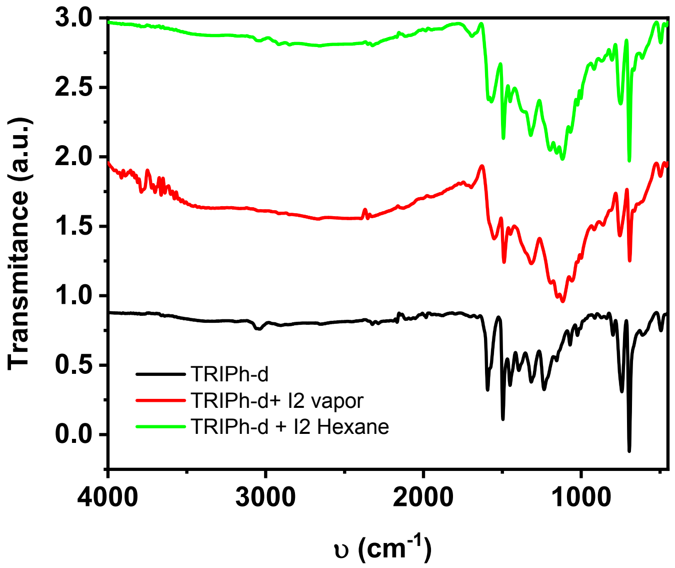

Figure S17. FT-IR spectra of **TRIPh-d** before (black) and after (red) adsorption of Iodine.

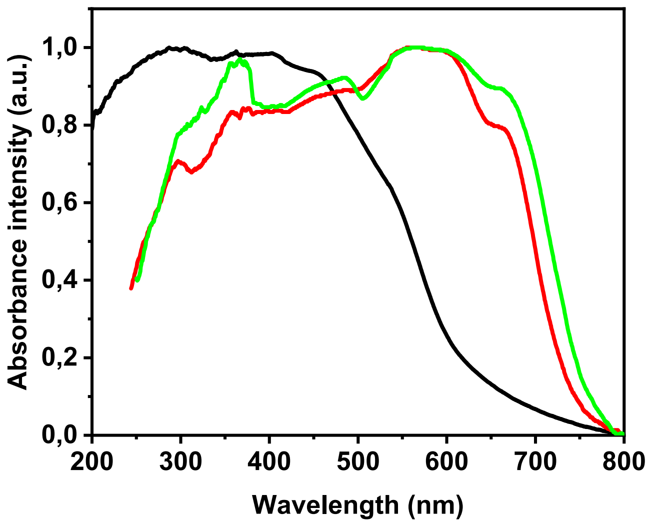

Figure S18. Comparison of UV-vis solid spectra of **TRIPh-d** without iodine (black), iodine adsorption in vapor (red) and in hexane (green).

8. Electrochemical measurements

8.1 Preparation of iodine-loaded polymer for electrochemical measurements

100 mg of polymer was suspended in a n-hexane solution of iodine (900 mg/mL) for 48h. After this time the suspension was filtered and a black solid was obtained. The iodine load for **TRIPh** POPs was determined by TGA.

8.2 Evaluation of iodine-loaded TRIPh-d as cathode in Zn/I_2 batteries.

For aqueous batteries, Swagelok cells were assembled under air condition. Zinc foil served as anode, 30 m $ZnCl_2$ as the electrolyte and PVDF filter paper as separator. For cathode preparation, 40 mg of the polymer with iodine was mixed in a mortar with 5 mg of carbon black using a small amount of isopropanol (IpOH), that acted like a dispersing agent. Once a homogeneous mixture was achieved, 5 mg of PTFE emulsion was added. The mixture was continuously stirred, with additional IpOH if necessary, to prevent drying, until a thick slurry was formed. This slurry was pressed and cut into circular disks of 8 mm of diameter, and weight of about 3 mg.

Charge storage analysis was conducted by performing CV measurements at various scan rates to evaluate the capacitive and diffusion-controlled contributions. The relation between peak current and scan rate is given by:

$$i = a\,v^b \qquad \text{Equation S7}$$

Where i is the peak current (mA), v is the scan rate (mV s^{-1}), and a and b are adjustable parameters. When the extracted value of b is close to 1 a capacitive-controlled process is described, while a diffusion-controlled process is related to a b value near 0.5. To quantify the capacitive and diffusion contributions (%) the total current response is related to scan rate using the next equation:

$$i = k_1 v + k_2 v^{1/2} \qquad \text{Equation S8}$$

Where i is the total current (mA), $k_1 v$ represents the capacitive contribution, and $k_2 v^{1/2}$ the diffusive contribution.

The capacity of each electrode based on the GCD data was calculated according to the mass of iodine using the following equation:

$$C = \frac{I}{m} \cdot \Delta t_d \qquad \text{Equation S9}$$

Where C is the specific capacity of the electrode (mA h g^{-1}), I is the applied current (mA), Δt_d is discharge time (h), m is the mass of each electrode (g).

The Coulombic efficiency (CE) was calculated according to the next equation:

$$CE = \frac{\Delta t_d}{\Delta t_c} \qquad \text{Equation S10}$$

Where Δt_d is discharge time (h) and Δt_c is charge time (h).

Energy density of the electrodes was calculated by the next equation:

$$E = \frac{V \cdot C}{1000} \qquad \text{Equation S11}$$

Where E is the energy density (W h kg^{-1}), V is the discharge potential (V) and C is the specific capacity (mA h g^{-1}).

Power density can be determined from E as so:

$$P = \frac{E}{\Delta t_d} \qquad \text{Equation S12}$$

Where P is power density (W kg^{-1}) and Δt_d is discharge time (h).

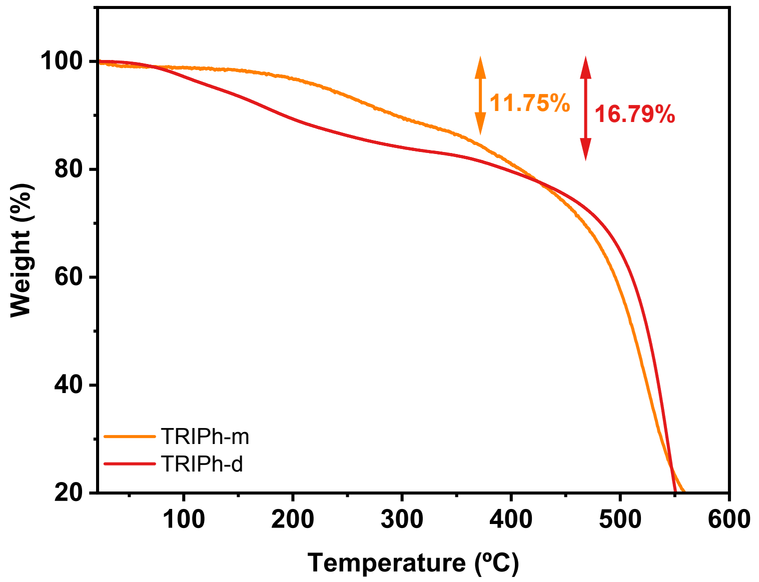

Figure S19. TGA curves performed under air of iodine loaded **TRIPh-d** powders.

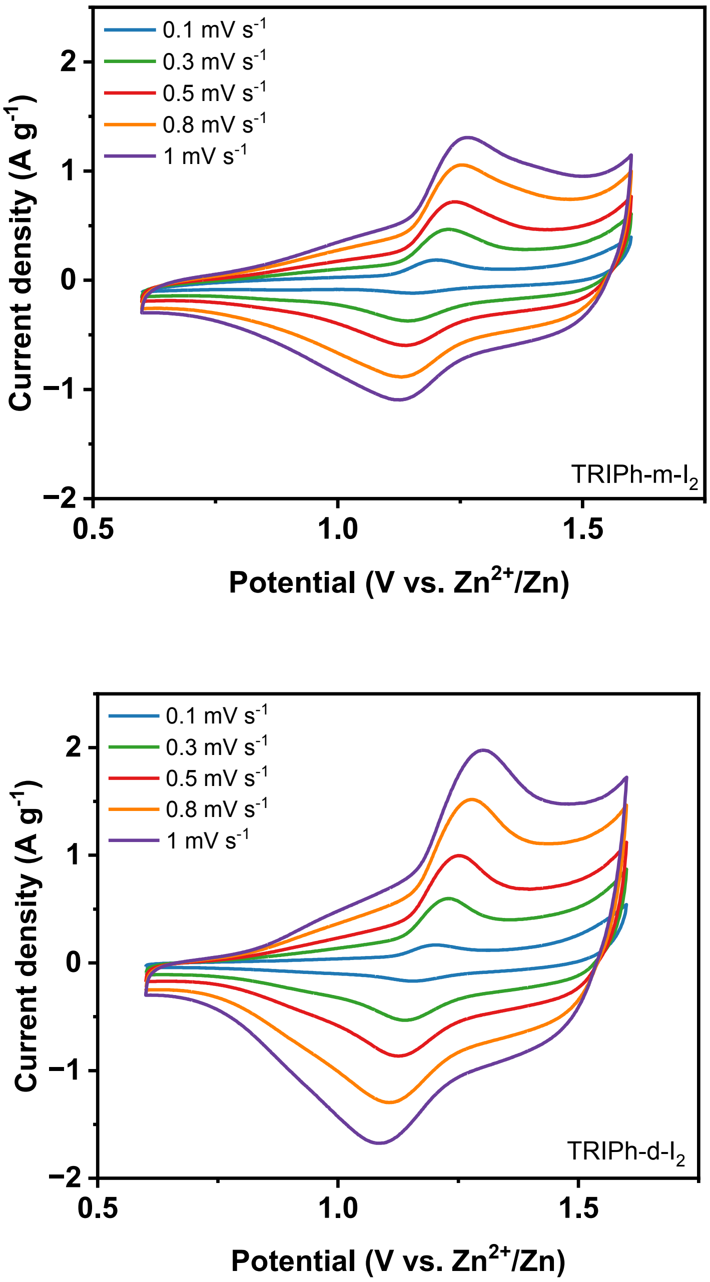

Figure S20. CVs at different scan rates of **TRIPh-d** and **TRIPh-m.**

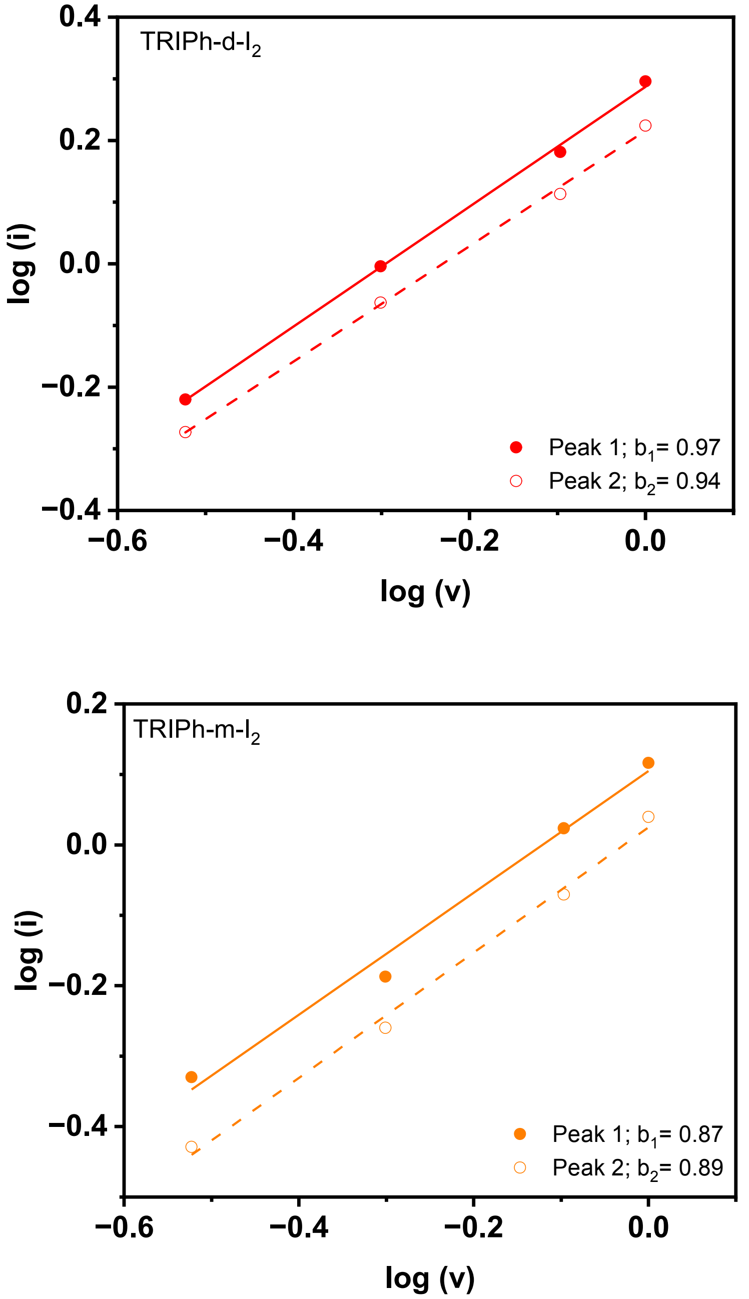

Figure S21. Log (i) vs log (v) plot of **TRIPh-d** and **TRIPh-m.** Peak 1 corresponds to the oxidation process and peak 2 to the reduction.

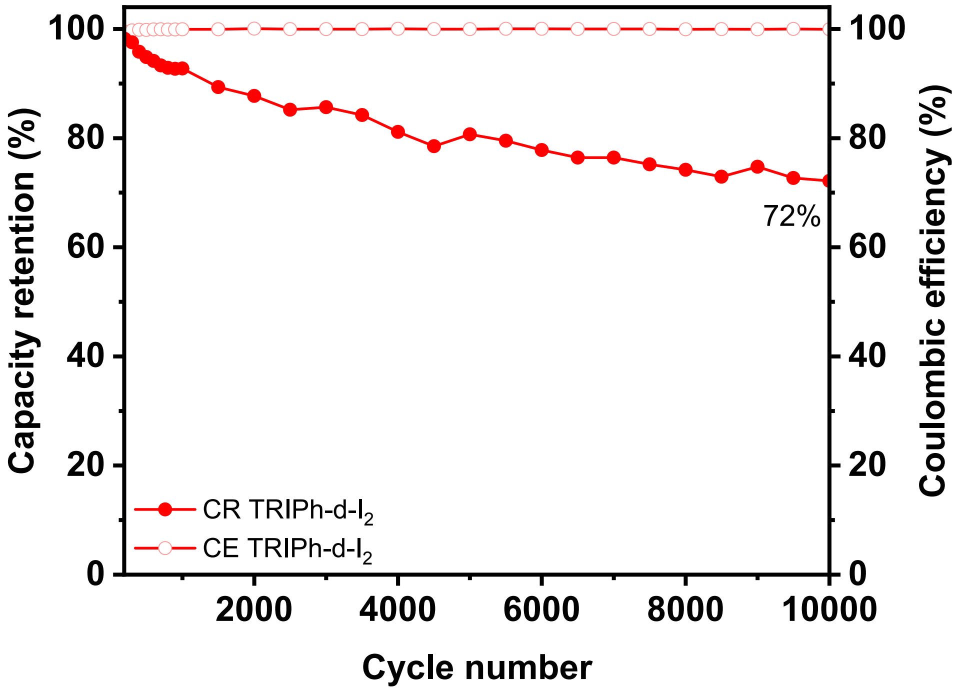

Figure S22. Capacity retention and Coulombic efficiency during cycling at 5 A g^{-1} of **TRIPh-d** based ZIB.

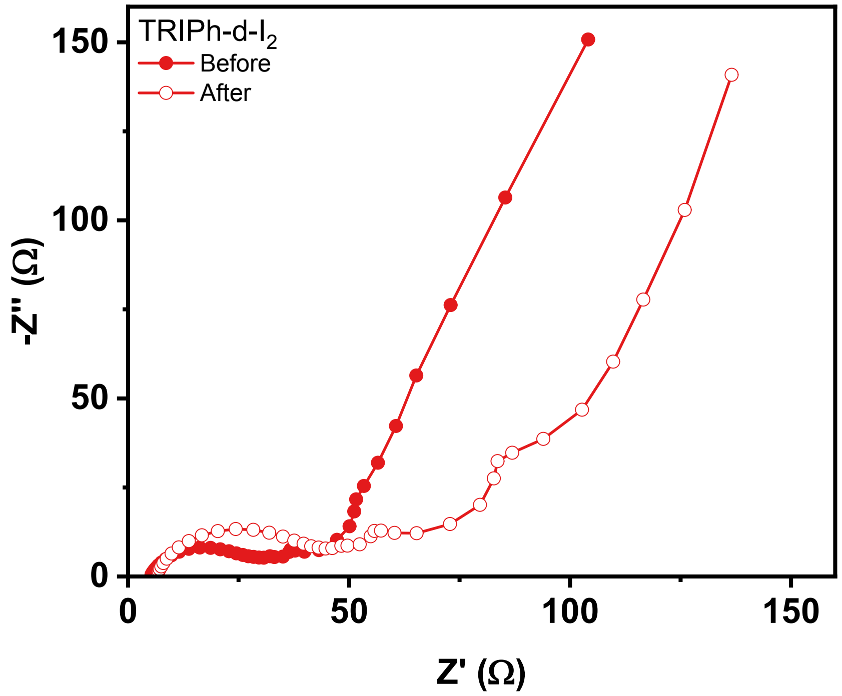

Figure S23. Nyquist plot of the Zn/I_2 **TRIPh-d** cell before and after 2000 cycling at 5 A g^{-1}.

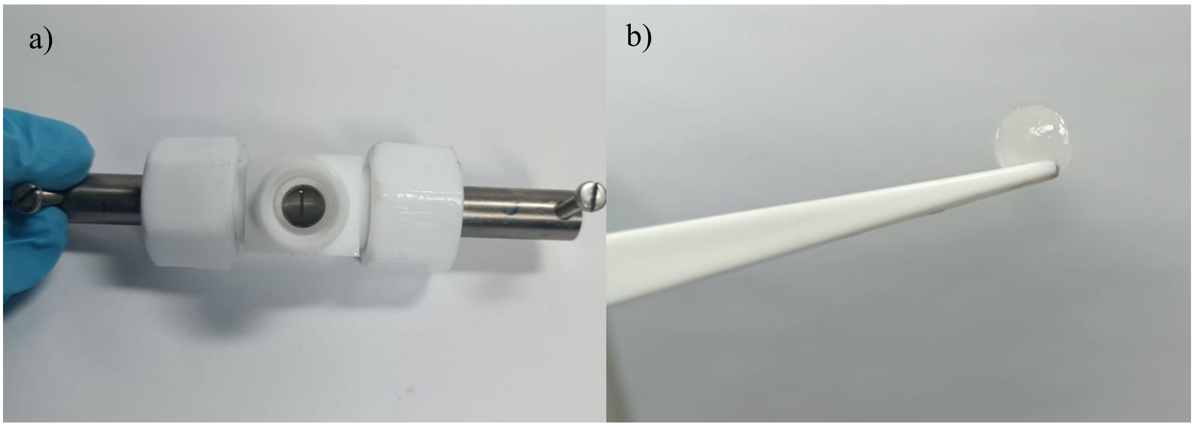

Figure S24: (a) Digital photograph of the Zn/I_2 **TRIPh-d** cell after 2000 cycling at 5 A g^{-1}, showing a transparent, non-colored electrolyte. (b) Digital photograph of the PVDF separator from the Zn/I_2 **TRIPh-d** cell after cycling at 5 A g^{-1}, exhibiting a clean, non-colored appearance.

Table S8. Comparison of rate, capacity and capacity retention of this work with recent Zn-I_2 systems based on iodine-capturing porous organic and carbon-based materials.

Cathode	Cycle number (Rate)	Capacity (mA h g^{-1})	Capacity retention (%)	Energy density (W h kg^{-1})	Power density (W kg^{-1})	Ref
**TRIPh-d**	500 (1 A g^{-1})<br>10000 (5 A g^{-1})	228 (1 A g^{-1})	96 (1 A g^{-1})<br>72 (5 A g^{-1})	228	1000	This work
**I_2@PAF-1**	20000 (10C)	328 (0,5C)	86	271	2260	[33]
**I^-/I^{-3}@Zn-TCPP**	5000 (5 A g^{-1})	278 (5 A g^{-1})	98	340	-	[34]
**PANI-I_2**	700 (1,5 A g^{-1})	160 (1,5 A g^{-1})	79	-	-	[35]
**ZPC/I_2**	1000 (0,8 A g^{-1})	156 (1 A g^{-1})	72	-	-	[36]
**ACC/I_2**	1500 (5C)	255 (5C)	90	151	-	[37]
**CC/I_2**	200 (2 mA cm^{-2})	355 (1 mA cm^2)	91	400	-	[38]
**AC@I_2**	10000 (5 A g^{-1})	210 (0,1 A g^{-1})	66	237.4	112	[39]
**ACF@I_2**	3000 (2C)	174 (1C)	90	-	-	[40]
**NCCs@I_2**	1000 (0,1 A g^{-1})	259 (0,1 A g^{-1})	100	282	-	[41]
**HC@FeNC**	60000 (10 A g^{-1})	300 (10 A g^{-1})	88	253	-	[42]
**Starch/I_2**	2000 (0.2 A g^{-1})<br>25000 (1 A g^{-1})	132.2 (0.2 A g^{-1})	88 (1 A g^{-1})	2500	80	[43]
**AC/I_2**	50000 (10C)<br>2000 (2C)	125 (10C)<br>243.5 (2C)	90 (10C)<br>95 (2C)	-	-	[44]
**CCTF TPMB**	30000 (5 A g^{-1})	180 (5 A g^{-1})	94	-	-	[45]
**FCOF**	500 (1C)<br>40000 (50C)	197 (1C)<br>113 (50C)	99.7<br>100	-	-	[46]
**CMK-3@I_2**	39000 (10 A g^{-1})	90 (5 A g^{-1})	81	150	250	[47]

9. References

(1) Beamson, G.; Briggs, D. *High Resolution XPS of Organic Polymers: The Scienta ESCA300 Database*; Wiley, 1992.

(2) Wang, L.; Fang, Q.; Lu, Q.; Zhang, S.; Jin, Y.; Liu, Z. Octupolar (C_3 and S_4) Symmetric Cyclized Indole Derivatives: Syntheses, Structures, and NLO Properties. *Org. Lett.* **2015**, *17* (17), 4164–4167. https://doi.org/10.1021/acs.orglett.5b01912.

(3) Guadalupe, J.; Ray, A. M.; Maya, E. M.; Gómez-Lor, B.; Iglesias, M. Truxene-Based Porous Polymers: From Synthesis to Catalytic Activity. *Polym. Chem.* **2018**, *9* (36), 4585–4595. https://doi.org/10.1039/C8PY01082J.

(4) Echeverri, M.; Gámez-Valenzuela, S.; González-Cano, R. C.; Guadalupe, J.; Cortijo-Campos, S.; López Navarrete, J. T.; Iglesias, M.; Ruiz Delgado, M. C.; Gómez-Lor, B. Effect of the Linkage Position on the Conjugation Length of Truxene-Based Porous Polymers: Implications for Their Sensing Performance of Nitroaromatics. *Chem. Mater.* **2019**, *31* (17), 6971–6978. https://doi.org/10.1021/acs.chemmater.9b01432.

(5) Farooq, N.; Malik, M. A.; Hashmi, A. A. Effective Iodine Adsorption and Storage of Volatile Iodine by Nitrogen-Rich Porous Organic Polymers from Flexible Building Blocks. *ACS Appl. Polym. Mater.* **2024**, *6* (13), 7368–7382. https://doi.org/10.1021/acsapm.4c00187.

(6) Bera, S.; Sau, S.; Banerjee, F.; Kumar, N.; Samanta, S. K. Phosphate-Based, Heteroatom-Rich Porous Organic Polymers for Efficacious Uptake of Iodine in Vapor Phase. *Separation and Purification Technology* **2025**, *352*, 128123. https://doi.org/10.1016/j.seppur.2024.128123.

(7) Qiu, N.; Wang, H.; Tang, R.; Yang, Y.; Kong, X.; Hu, Z.; Zhong, F.; Tan, H. Synthesis of Phenothiazine-Based Porous Organic Polymer and Its Application to Iodine Adsorption. *Microporous and Mesoporous Materials* **2024**, *363*, 112833. https://doi.org/10.1016/j.micromeso.2023.112833.

(8) Farooq, N.; Malik, M. A.; Taha, A.; Hashmi, A. A. Acridine-Based Nitrogen-Rich Porous Organic Polymer for Ultra-Fast Iodine Uptake and Reversible Storage of Iodine. *ACS Appl. Eng. Mater.* **2025**, *3* (8), 2354–2364. https://doi.org/10.1021/acsaenm.5c00276.

(9) Xu, Y.; Yu, H.; Shi, B.; Gao, S.; Zhang, L.; Li, X.; Liao, X.; Huang, K. Room-Temperature Synthesis of Hollow Carbazole-Based Covalent Triazine Polymers with Multiactive Sites for Efficient Iodine Capture-Catalysis Cascade Application. *ACS Appl. Polym. Mater.* **2020**, *2* (8), 3704–3713. https://doi.org/10.1021/acsapm.0c00582.

(10) Yang, J.; Wang, S.; Yan, Q.; Hu, H.; Xu, H.; Ma, H.; Su, X.; Gao, Y. Novel Nitrogen-Rich Conjugated Microporous Polymers for Efficient Capture of Iodine and Methyl Iodide. *Polym. Chem.* **2024**, *15* (26), 2652–2661. https://doi.org/10.1039/D4PY00425F.

(11) Shi, Z.; Tang, H.; Sun, L.; Wang, J.; Li, J.; Wang, D.; Qi, L.; Wang, L.; Dong, G.; Zhao, M. Benzonquanmine-Based Hypercrosslinked Polymers for High-Efficiency and Reversible Iodine Capture. *Separation and Purification Technology* **2025**, *359*, 130570. https://doi.org/10.1016/j.seppur.2024.130570.

(12) Altınışık, S.; Yayla, C.; Karaca, N.; Koyuncu, S. Carbazole–Bismaleimide Based Hyper-Cross-Linked Porous Organic Polymer for Efficient Iodine Capture. *Langmuir* **2025**, *41* (5), 3259–3268. https://doi.org/10.1021/acs.langmuir.4c04125.

(13) Junthod, K.; Todee, B.; Khamphaijun, K.; Chutimasakul, T.; Sangtawesin, T.; Ratvijitvech, T.; Tantirungrotechai, J.; Suriya, U.; Bunchuay, T. Halogen-Bonding Interaction-Mediated Efficient Iodine Capture of Highly Nitrogen-Functionalized Hyper-Crosslinked Polymers. *ACS Appl. Polym. Mater.* **2024**, acsapm.4c00931. https://doi.org/10.1021/acsapm.4c00931.

(14) Peng, Y.; Meng, T.; Lei, H.; Li, Z.; Chaleawlert-umpon, S.; Shen, M.; Yang, K.; Zhao, X.; Li, L. Enhanced Iodine Adsorption Performance of Porous Aromatic Frameworks

from Nitrogen-Containing Monomers. *Separation and Purification Technology* **2025**, *370*, 133230. https://doi.org/10.1016/j.seppur.2025.133230.

(15) Cui, B.; Bu, N.; Fu, Y.; Lu, J.; Liang, L.; Yao, W.; Wang, S.; Li, N.; Yang, L.; Yan, Z.; Yuan, Y.; Xia, L. Constructing a Phenyl Bridged Switch in Porous Aromatic Frameworks for Enhancing the Adsorption Capacity of Volatile Iodine. *Applied Surface Science* **2024**, *653*, 159422. https://doi.org/10.1016/j.apsusc.2024.159422.

(16) Ran, Y.; Yang, M.; Li, J.; Song, J.; Wang, Y.; Li, Z.; Yuan, L. Constructing Covalent Organic Frameworks with Dense Thiophene S Sites for Effective Iodine Capture. *Separation and Purification Technology* **2025**, *355*, 129603. https://doi.org/10.1016/j.seppur.2024.129603.

(17) Qi, S.; Yao, Y.; Han, Y.; You, C.; Yang, C.; Sun, D.; Jiang, W.; Ren, B.; Ma, Y.; Liu, C. Post-Synthesis Modification Cationic Covalent Organic Frameworks with Multiple Adsorption Sites for Efficient Iodine Adsorption. *Colloids and Surfaces A: Physicochemical and Engineering Aspects* **2024**, *702*, 135100. https://doi.org/10.1016/j.colsurfa.2024.135100.

(18) Ren, L.-Y.; Geng, T.-M. Constructing Cationic Flexible Covalent Organic Frameworks through Post-Functionalization for Enhancing the Iodine Adsorption Capacity. *Chemical Engineering Journal* **2024**, *480*, 148076. https://doi.org/10.1016/j.cej.2023.148076.

(19) Sun, H.; La, P.; Zhu, Z.; Liang, W.; Yang, B.; Li, A. Capture and Reversible Storage of Volatile Iodine by Porous Carbon with High Capacity. *J Mater Sci* **2015**, *50* (22), 7326–7332. https://doi.org/10.1007/s10853-015-9289-1.

(20) Tang, Z.; Xie, D.; Li, S.; Huang, L. The Directions of Enhanced Activated Carbon Fibers for Iodine Capture from Humid Gas Streams: Synergistic Mechanisms of Microporosity, Hydrophobicity, and Nitrogen Sites. *Separation and Purification Technology* **2025**, *354*, 129032. https://doi.org/10.1016/j.seppur.2024.129032.

(21) Zheng, W.; Huang, J.; Tian, Z.; Yang, Z.; Leng, L.; He, W.; Chen, J.; Zeng, X.; Yang, W.; Qu, W.; Li, H. Metal Sulfide Functionalized Activated Carbon for Efficient Capture of Gaseous Iodine. *Chemical Engineering Science* **2025**, *303*, 120955. https://doi.org/10.1016/j.ces.2024.120955.

(22) Wu, Y.; Zuo, Q.; Jiang, T.; Wang, Z.; Pan, J. H.; Ji, Z. Design and Fabrication of Phosphazene-Based Porous Organic Materials for Iodine Adsorption. *Environ. Sci.: Nano* **2025**, 10.1039.D5EN00136F. https://doi.org/10.1039/D5EN00136F.

(23) Khosravi Esmaeiltarkhani, F.; Dinari, M.; Mokhtari, N. Nitrogen-Rich Porous Organic Polymer as a Promising Adsorbent for Iodine Capture from Organic Solvents. *New J. Chem.* **2024**, *48* (5), 1943–1951. https://doi.org/10.1039/D3NJ04674E.

(24) Zhao, Y.; Lu, W.; Zhang, Y.; Liu, X.; Sun, B. Room Temperature Synthesis of Piperazine-Based Nitrogen-Rich Porous Organic Polymers for Efficient Iodine Adsorption. *Microporous and Mesoporous Materials* **2024**, *366*, 112954. https://doi.org/10.1016/j.micromeso.2023.112954.

(25) Anand, A.; Bhagat, R. K.; Ghosh, S.; Chattopadhyay, S. Triazine-Tryptophan Based Mesoporous Polymer: Ultrafast Synthesis in a Minute and Efficient Removal of Iodine. *ACS Appl. Polym. Mater.* **2024**, *6* (18), 11487–11496. https://doi.org/10.1021/acsapm.4c02140.

(26) Zheng, Q.; Huang, B.; Du, X.; Zhang, J.; Fu, H.; Gao, H.; Liao, Y. Construction of N-Loaded Conjugated Polymer for Highly Effective Removal of Iodine in Organic Solution. *Journal of Environmental Chemical Engineering* **2023**, *11* (1), 109125. https://doi.org/10.1016/j.jece.2022.109125.

(27) Sarkar, S.; Ghosh, T.; Chakrobarty, A.; Majhi, J.; Nag, P.; Bandyopadhyay, A.; Vennapusa, S. R.; Kumar, R.; Mukhopadhyay, S. Exploring a Redox-Active Ionic Porous Organic Polymer in Environmental Remediation and Electrochromic Application. *ACS*

Appl. Mater. Interfaces **2023**, *15* (23), 28453–28464. https://doi.org/10.1021/acsami.3c01800.

(28) Lu, W.-J.; Zhao, Y.-Y.; Sun, B.-W. Nitrogen-Rich Nonporous Covalent Organic Polymers for Highly Efficient and Reversible Iodine Capture. *Journal of Solid State Chemistry* **2024**, *336*, 124724. https://doi.org/10.1016/j.jssc.2024.124724.

(29) Zhu, H.; Qin, Y.; Guo, Y.; Shen, Z.; Imran, M.; Asim Mushtaq, M.; Zhang, Z.; Ni, C.; Chen, Y.; Ding, Y.; Gul, H.; Zou, J.; Tsiakaras, P.; Hsu, H.-Y.; Zhao, J. Covalent Organic Polymers for Efficient Removal of Iodine from Gas- and Liquid-Phase Environments. *Chemical Engineering Journal* **2024**, *484*, 149739. https://doi.org/10.1016/j.cej.2024.149739.

(30) Li, Y.; Zhang, J.; Wang, X.; Meng, R.; Zhang, Y.; Yin, L.; Pi, X.; Du, Q.; Chen, L.; Li, Y. Amino Functionalization of Covalent Organic Frameworks for Enhancing Adsorption Capacity of Radioactive Iodine. *Chemical Engineering Journal* **2025**, *513*, 163043. https://doi.org/10.1016/j.cej.2025.163043.

(31) Ruidas, S.; Chowdhury, A.; Ghosh, A.; Ghosh, A.; Mondal, S.; Wonanke, A. D. D.; Addicoat, M.; Das, A. K.; Modak, A.; Bhaumik, A. Covalent Organic Framework as a Metal-Free Photocatalyst for Dye Degradation and Radioactive Iodine Adsorption. *Langmuir* **2023**, *39* (11), 4071–4081. https://doi.org/10.1021/acs.langmuir.2c03379.

(32) Ma, J.; Xu, S.; Wang, X.; Zhang, M.; Qu, Y.; Cao, Q.; Jia, H.; Xu, J.; Wang, X. Biomass Derived Porous Carbon for Efficient Iodine Adsorption from Vapor and Solution. *Separation and Purification Technology* **2024**, *347*, 127613. https://doi.org/10.1016/j.seppur.2024.127613.

(33) Hu, J.; Zhang, Z.; Deng, T.; Cui, F. C.; Shi, X.; Tian, Y.; Zhu, G. Porous Aromatic Frameworks Enabling Polyiodide Confinement toward High Capacity and Long Lifespan Zinc–Iodine Batteries. *Advanced Materials* **2024**, *36* (29), 2401091. https://doi.org/10.1002/adma.202401091.

(34) Tan, Y.; Chen, Z.; Tao, Z.; Wang, A.; Lai, S.; Yang, Y. A Two-Dimensional Porphyrin Coordination Supramolecular Network Cathode for High-Performance Aqueous Dual-Ion Battery. *Angew Chem Int Ed* **2023**, *62* (12), e202217744. https://doi.org/10.1002/anie.202217744.

(35) Zeng, X.; Meng, X.; Jiang, W.; Liu, J.; Ling, M.; Yan, L.; Liang, C. Anchoring Polyiodide to Conductive Polymers as Cathode for High-Performance Aqueous Zinc–Iodine Batteries. *ACS Sustainable Chem. Eng.* **2020**, *8* (38), 14280–14285. https://doi.org/10.1021/acssuschemeng.0c05283.

(36) Xu, J.; Wang, J.; Ge, L.; Sun, J.; Ma, W.; Ren, M.; Cai, X.; Liu, W.; Yao, J. ZIF-8 Derived Porous Carbon to Mitigate Shuttle Effect for High Performance Aqueous Zinc–Iodine Batteries. *Journal of Colloid and Interface Science* **2022**, *610*, 98–105. https://doi.org/10.1016/j.jcis.2021.12.043.

(37) Bai, C.; Cai, F.; Wang, L.; Guo, S.; Liu, X.; Yuan, Z. A Sustainable Aqueous Zn-I2 Battery. *Nano Res.* **2018**, *11* (7), 3548–3554. https://doi.org/10.1007/s12274-017-1920-9.

(38) Li, Y.; Liu, L.; Li, H.; Cheng, F.; Chen, J. Rechargeable Aqueous Zinc–Iodine Batteries: Pore Confining Mechanism and Flexible Device Application. *Chem. Commun.* **2018**, *54* (50), 6792–6795. https://doi.org/10.1039/C8CC02616E.

(39) Li, W.; Wang, K.; Jiang, K. A High Energy Efficiency and Long Life Aqueous Zn–I_2 Battery. *J. Mater. Chem. A* **2020**, *8* (7), 3785–3794. https://doi.org/10.1039/C9TA13081K.

(40) Pan, H.; Li, B.; Mei, D.; Nie, Z.; Shao, Y.; Li, G.; Li, X. S.; Han, K. S.; Mueller, K. T.; Sprenkle, V.; Liu, J. Controlling Solid–Liquid Conversion Reactions for a Highly Reversible Aqueous Zinc–Iodine Battery. *ACS Energy Lett.* **2017**, *2* (12), 2674–2680. https://doi.org/10.1021/acsenergylett.7b00851.

(41) Liu, W.; Liu, P.; Lyu, Y.; Wen, J.; Hao, R.; Zheng, J.; Liu, K.; Li, Y.-J.; Wang, S. Advanced Zn–I_2 Battery with Excellent Cycling Stability and Good Rate Performance by a Multifunctional Iodine Host. *ACS Appl. Mater. Interfaces* **2022**, *14* (7), 8955–8962. https://doi.org/10.1021/acsami.1c21026.
(42) Wang, Y.; Jin, X.; Xiong, J.; Zhu, Q.; Li, Q.; Wang, R.; Li, J.; Fan, Y.; Zhao, Y.; Sun, X. Ultrastable Electrolytic Zn–I_2 Batteries Based on Nanocarbon Wrapped by Highly Efficient Single-Atom Fe-NC Iodine Catalysts. *Advanced Materials* **2024**, *36* (30), 2404093. https://doi.org/10.1002/adma.202404093.
(43) Gao, Y. Integrated Confinement-Chemisorption-Catalysis Cathode for Highly Stable Zinc-Iodine Batteries.
(44) Liu, T.; Lei, C.; Yang, W.; Wang, H.; Ma, W.; Li, J.; Liang, X. Solvent Chemistry Manipulated Iodine Redox Thermodynamics For Durable Iodine Batteries. *Angew Chem Int Ed* **2025**, *64* (18), e202422163. https://doi.org/10.1002/anie.202422163.
(45) Zhao, Y.; Wang, Y.; Xue, W.; Cheng, R.; Zheng, X.; Zhu, G.; Hu, D.; Huang, H.; Hu, C.; Liu, D. Unveiling the Role of Cationic Pyridine Sites in Covalent Triazine Framework for Boosting Zinc–Iodine Batteries Performance. *Advanced Materials* **2024**, *36* (31), 2403097. https://doi.org/10.1002/adma.202403097.
(46) Tan, L.; Wei, J.; Xue, L.; Luo, D.; Chen, G.; Chu, Y.; Wang, J.; Zong, K.; Song, X.; Yang, L.; Wang, X.; Chen, Z. Regulating the Electron Structure of Covalent Organic Frameworks to Enable Excellent Cycle Life and High Rate toward Advanced Zn−I_2 Batteries. *Adv Funct Materials* **2025**, *35* (13), 2416931. https://doi.org/10.1002/adfm.202416931.
(47) Guo, Q.; Wang, H.; Sun, X.; Yang, Y.; Chen, N.; Qu, L. In Situ Synthesis of Cathode Materials for Aqueous High-Rate and Durable Zn–I_2 Batteries. *ACS Materials Lett.* **2022**, *4* (10), 1872–1881. https://doi.org/10.1021/acsmaterialslett.2c00608.
